# Supplementary material for: Study protocol: a multicentre, open-label, parallel-group, phase 2, randomised controlled trial of autologous macrophage therapy for liver cirrhosis (MATCH)
Source: BMJ Open. 2021 Nov 8;11(11):e053190. doi: 10.1136/bmjopen-2021-053190 (PMC8576470; doi:10.1136/bmjopen-2021-053190)
Supplement: Author's manuscript [file bmjopen-2021-053190.draft_revisions.pdf]

BMJ Open is committed to open peer review. As part of this commitment we make the peer review history of every article we publish publicly available.

When an article is published we post the peer reviewers' comments and the authors' responses online. We also post the versions of the paper that were used during peer review. These are the versions that the peer review comments apply to.

The versions of the paper that follow are the versions that were submitted during the peer review process. They are not the versions of record or the final published versions. They should not be cited or distributed as the published version of this manuscript.

BMJ Open is an open access journal and the full, final, typeset and author-corrected version of record of the manuscript is available on our site with no access controls, subscription charges or pay-per-view fees (http://bmjopen.bmj.com).

If you have any questions on BMJ Open's open peer review process please email
info.bmjopen@bmj.com

BMJ Open

Study Protocol: A multicentre, open-label, parallel-group, phase 2, randomised controlled trial of autologous macrophage therapy for liver cirrhosis (MATCH)

Journal:	*BMJ Open*
Manuscript ID	bmjopen-2021-053190
Article Type:	Protocol
Date Submitted by the Author:	06-May-2021
Complete List of Authors:	Brennan, Paul; The University of Edinburgh Edinburgh Medical School, Centre for Regenerative Medicine<br>MacMillan, Mark; The University of Edinburgh Edinburgh Medical School<br>Manship, Thomas; NHS Lothian, CLDD<br>Moroni, Francesca; NHS Grampian<br>Glover, Alison; Scottish National Blood Transfusion Service<br>Graham, Catriona; The University of Edinburgh<br>Semple, Scott; The University of Edinburgh Deanery of Clinical Sciences, Centre for Cardiovascular Science<br>Morris, David; The University of Edinburgh Deanery of Clinical Sciences, Centre for Cardiovascular Science<br>Fraser, Alasdair; SNBTS, Tissues, Cells and Advanced Therapeutics<br>Pass, Chloe; SNBTS, Tissues, Cells and Advanced Therapeutics<br>McGowan, Neil; SNBTS, Tissues, Cells and Advanced Therapeutics<br>Turner, Marc; SNBTS, Tissues, Cells and Advanced Therapeutics<br>Lachlan, Neil; NHS Greater Glasgow and Clyde<br>Dillon, John; University of Dundee Division of Cardiovascular and Diabetes Medicine, Liver Group<br>Campbell, John; SNBTS, Tissues, Cells and Advanced Therapeutics<br>Fallowfield, Jonathan; University of Edinburgh MRC Centre for Inflammation Research, Queen's Medical Research Institute<br>Forbes, Stuart; The University of Edinburgh Deanery of Clinical Sciences, Centre for Regenerative Medicine
Keywords:	Hepatobiliary disease < GASTROENTEROLOGY, Immunology < NATURAL SCIENCE DISCIPLINES, Clinical trials < THERAPEUTICS, Cell biology < NATURAL SCIENCE DISCIPLINES

SCHOLARONE™
Manuscripts

I, the Submitting Author has the right to grant and does grant on behalf of all authors of the Work (as defined in the below author licence), an exclusive licence and/or a non-exclusive licence for contributions from authors who are: i) UK Crown employees; ii) where BMJ has agreed a CC-BY licence shall apply, and/or iii) in accordance with the terms applicable for US Federal Government officers or employees acting as part of their official duties; on a worldwide, perpetual, irrevocable, royalty-free basis to BMJ Publishing Group Ltd ("BMJ") its licensees and where the relevant Journal is co-owned by BMJ to the co-owners of the Journal, to publish the Work in this journal and any other BMJ products and to exploit all rights, as set out in our licence.

The Submitting Author accepts and understands that any supply made under these terms is made by BMJ to the Submitting Author unless you are acting as an employee on behalf of your employer or a postgraduate student of an affiliated institution which is paying any applicable article publishing charge ("APC") for Open Access articles. Where the Submitting Author wishes to make the Work available on an Open Access basis (and intends to pay the relevant APC), the terms of reuse of such Open Access shall be governed by a Creative Commons licence – details of these licences and which Creative Commons licence will apply to this Work are set out in our licence referred to above.

Other than as permitted in any relevant BMJ Author's Self Archiving Policies, I confirm this Work has not been accepted for publication elsewhere, is not being considered for publication elsewhere and does not duplicate material already published. I confirm all authors consent to publication of this Work and authorise the granting of this licence.

For peer review only

Study Protocol: A multicentre, open-label, parallel-group, phase 2, randomised controlled trial of autologous macrophage therapy for liver cirrhosis (MATCH)

Paul N Brennan[1,3*], Mark MacMillan[1], Thomas Manship[3], Francesca Moroni[5], Alison Glover[2], Catriona Graham[4], Scott IK Semple[6], David M Morris[6], Alasdair R Fraser[2], Chloe Pass[2], Neil WA McGowan[2], Marc L Turner[2], Neil Lachlan[7], John F Dillon[8], John DM Campbell[2], Jonathan A Fallowfield[3,10], Stuart J Forbes[1,3]

1. Centre for Regenerative Medicine, University of Edinburgh, Edinburgh, UK
2. Scottish National Blood Transfusion Service (SNBTS), Edinburgh, UK
3. Centre for Liver and Digestive Disorders, Royal Infirmary of Edinburgh, Edinburgh, UK
4. Wellcome Trust Clinical Research Facility, University of Edinburgh, Edinburgh, UK
5. Aberdeen Royal Infirmary, NHS Grampian, Foresterhill Health Campus, Aberdeen, UK
6. The University of Edinburgh Deanery of Clinical Sciences, Centre for Cardiovascular Science, Edinburgh, UK
7. The University of Edinburgh Deanery of Clinical Sciences, Edinburgh Imaging Edinburgh, UK
8. NHS Greater Glasgow and Clyde, Glasgow Royal Infirmary, Glasgow, UK
9. University of Dundee, Ninewells Hospital and Medical School, Dundee, UK
10. Centre for Inflammation Research, University of Edinburgh, Edinburgh, UK

*Corresponding author: Pbrenna2@ed.ac.uk

Word Count: 4,484 (Excl. References and Tables)

For peer review only

Abstract

Introduction

Liver cirrhosis is a growing global healthcare challenge. Cirrhosis is characterized by severe liver fibrosis, organ dysfunction and complications related to portal hypertension. There are no licensed antifibrotic or pro-regenerative medicines and liver transplantation is a scarce resource. Hepatic macrophages can promote both liver fibrogenesis and fibrosis regression. The safety and feasibility of peripheral infusion of ex vivo matured autologous monocyte-derived macrophages in patients with compensated cirrhosis has been demonstrated.

Methods and Analysis

The efficacy of autologous macrophage therapy, compared to standard medical care, will be investigated in a cohort of adult patients with compensated cirrhosis in a multicentre, open-label, parallel-group, phase 2, randomised controlled trial. The primary outcome is the change in Model for End-Stage Liver Disease (MELD) score at 90 days. The trial will provide the first high-quality examination of the efficacy of autologous macrophage therapy in improving liver function, non-invasive fibrosis markers and other clinical outcomes in patients with compensated cirrhosis.

Ethics and dissemination

The trial will be conducted according to the ethical principles of the Declaration of Helsinki 2013 and has been approved by Scotland A Research Ethics Committee (reference 15/SS/0121), NHS Lothian Research and Development department and the Medicine and Health Care Regulatory Agency (MHRA-UK). Final results will be presented in peer-reviewed journals and at relevant conferences.

Trial registration

The trial was registered prospectively in the International Standard Randomized Controlled Trial Number (ISRCTN) Registry (ISRCTN10368050) and European Union Clinical Trials Register (EudraCT; reference 2015-000963-15).

For peer review only

Protocol V14 – July 2020

Funding Statement

This work was supported by a Medical Research Council UK grant (Biomedical Catalyst Major Awards Committee, Reference: MR/M007588/1) to Prof. Stuart Forbes

Keywords: *Liver cirrhosis, macrophages, cell therapy, liver fibrosis, liver regeneration*

For peer review only

Strengths and limitations of this study

- Novel cellular-based therapy for liver cirrhosis.
- Well considered, varied assessments of markers of fibrosis
- Complementary assessment of quality of life indicators in those with chronic liver disease and potential benefit of being in a clinical trial
- Unblinded trial clinician, but all other aspects of trial blinded to investigators

For peer review only

Introduction

Liver disease is responsible for almost 2 million deaths per year globally, 1 million directly relating to complications of end-stage liver failure (ESLF) and a further 1 million due complications of hepatitis including hepatocellular carcinoma (HCC).[1] Cirrhosis and liver cancer are now respectively the 11th and 16th most common cause of death globally, accounting for 3.5% of all deaths. Variation in liver disease epidemiology occurs relative to the prevalence of modifiable risk factors including; harmful alcohol ingestion, obesity/metabolic syndrome and viral hepatitis.[2] There were 10·6 million prevalent cases of decompensated cirrhosis and 112 million prevalent cases of compensated cirrhosis globally in 2017.[3]

Cirrhosis represents the end-stage of chronic liver injury and progressive fibrosis (scarring), irrespective of the underlying aetiology. It is characterised by severe liver fibrosis leading to architectural disruption, hepatocyte dysfunction and portal hypertension. Cirrhosis typically affects those of working age, which has broad socio-economic impacts. Further, cirrhosis impairs health-related quality of life (HRQoL) whether due to mental impairment or limitations affecting the functioning of activities of daily living[4]; those with primary biliary cholangitis (PBC), non-alcoholic fatty liver disease (NAFLD) and hepatitis C virus (HCV) appear disproportionately affected.[5]

The classical dichotomy of chronic liver disease staging is compensated (asymptomatic) or decompensated cirrhosis. Acute decompensation delineates the development of one or more associated sequelae and is a key prognostic inflection point. The transition from compensated to decompensated cirrhosis occurs at a rate of about 5-7% per year.[6] Decompensation represents a prognostic milestone as it significantly alters mortality, with a cumulative 1 year mortality of 77% for those with stage 3 and 4 decompensated disease vs 4.4% in those with compensated disease. Importantly, emergency hospitalisation for decompensated liver disease heralds a deterioration in a patient's prognosis independent of stage of cirrhosis.[7]

Cirrhosis decompensation heralds the development of widespread organ dysregulation, including portal hypertension, splanchnic vasodilation, left ventricular impairment and systemic immune dysfunction. Inflammatory mediators of liver disease may underpin and potentiate nitric oxide-mediated capillary dysfunction, direct immunocytopathy and induce significant metabolic derangement, and redistribution of essential nutrient precursors.[8]

For patients in whom disease-specific therapy is unsuccessful or not possible, treatment options remain limited. Presently, although numerous agents have been evaluated in clinical trials, there are no approved pharmacological therapies for reversing fibrosis or stimulating liver regeneration in the cirrhotic liver.[9] Liver transplantation remains the only curative option for those with end-stage cirrhosis or HCC. Unfortunately, a significant proportion of those referred for transplant assessment are ineligible and ~12% die annually while on the waiting list in the UK.[10,11] Those who do undergo liver transplantation require lifelong immunosuppression with inherent risks of toxicity and adverse effects.[12]

Although whole organ or split liver transplantation are well established procedures to reinstate liver functional capacity, cell-based transplantation approaches are emerging.[13] Successful cell therapy could theoretically overcome organ availability limitations, whilst avoiding invasive surgical interventions. Successful hepatocyte transplantation involves reconstitution of as little as 1- 2.5% of functional tissue across a range of inherited metabolic liver diseases, and highlights the utility of such approaches.[14] Furthermore, there is a requirement for treatments that can 'bridge' patients with cirrhosis until a donor organ is available or allow spontaneous regeneration to occur following acute liver failure (ALF). Cell therapies that sufficiently remodel cirrhosis by reducing fibrosis and stimulating liver regeneration may also promote endogenous tissue repair and regeneration such that the need for transplantation is delayed or obviated.

For peer review only - http://bmjopen.bmj.com/site/about/guidelines.xhtml

Previous studies have typically focussed on the use of mesenchymal stromal cells (MSCs) or un-purified and heterogenous cell populations which will include pro-inflammatory and pro-fibrotic cell lineages. Despite promising preclinical studies, randomized controlled trials of autologous cell therapies in cirrhosis have to-date been disappointing.[15,16]

Macrophages are a heterogeneous, highly plastic population of cells with a diverse spectrum of roles within the liver including phagocytosis, maintaining immune tolerance and both promotion and resolution of inflammation and fibrosis (REF- use one of many reviews). During fibrogenesis macrophage-derived cytokines activate scar-producing hepatic stellate cells, whereas during fibrosis resolution macrophages can facilitate degradation of scar through the production of proteolytic enzymes.[17,18] Preclinical data demonstrated that autologous macrophage therapy improved liver function by stimulating fibrosis regression and augmenting liver regeneration in rodent models of advanced fibrosis.[19–21] We recently demonstrated the feasibility of performing apheresis in cirrhotic patients and differentiating autologous bone marrow derived monocytes into macrophages utilising GMP-compliant methods, reagents and equipment.[22] Moreover, in a first-in-human study we confirmed the safety, feasibility and maximum achievable dose of autologous macrophages. The study was not controlled and therefore unable to evaluate efficacy, however we observed some initial signals related to fibrosis remodelling and liver function that we wished to assess in a randomised study.[23]

Objectives

The primary objective of this phase 2 randomised controlled trial is to evaluate whether there is an improvement in liver function at 3 months in patients receiving autologous macrophage therapy compared to standard medical care.

The secondary objectives are to assess any improvement in markers of liver fibrosis, increased disease related quality of life, reduced liver related clinical events and prolonged transplant-free survival.

For peer review only

Trial Design

The MATCH trial is designed as a multicentre, open-label, parallel-group, phase 2, randomised controlled trial to compare autologous macrophage therapy with standard medical care in patients with compensated cirrhosis. Randomization will be performed with a 1:1 allocation and the primary outcome is the baseline to 90-day change in MELD score. Initially, the proposed trial was designed to administer 3 infusions to those randomised to the treatment arm. It quickly became apparent that it would not be feasible to complete the trial at within the desired timeframe and so it was decided that a single infusion should be adopted. This was agreed with the trial steering committee (TSC), sponsor and data monitoring committee (DMC).

For peer review only

Methods

Study oversight

The MATCH 0.1 trial is an investigator-led study, funded by the Medical Research Council (reference MR/M007588/1) and sponsored by ACCORD (Academic and Clinical Central Office for Research and Development for NHS Lothian/University of Edinburgh).Trial oversight is also provided by a trial steering committee (TSC) and data monitoring committee (DMC), who are impartial around aspects of study design and logistics, but provide independent advice and interval safety analyses. The study started initially in 2016 and is likely to continue until latter end of 2022. All study-related documents were designed by the trial team with input from ACCORD, an independent statistician and the Scottish National Blood Transfusion Service (SNBTS) team. The trial will be conducted according to the ethical principles of the Declaration of Helsinki 2013 and has been approved by Scotland A Research Ethics Committee (reference 15/SS/0121), NHS Lothian Research and Development department and the Medicine and Health Care Regulatory Agency (MHRA-UK). The trial was registered in the International Standard Randomized Controlled Trial registry (ISRCTN10368050) and the European Clinical Trial Database (reference 2015-000963-15). Good Clinical Practice regulations will be followed and written informed consent will be obtained from all participants.

Study Setting

The MATCH trial is recruiting in 3 hepatology centres in Scotland: Royal Infirmary of Edinburgh (Tertiary Transplant Centre/Level 3 hepatology services), Ninewells Hospital, Dundee and Glasgow Royal Infirmary (both Level 2 hepatology centres). There are plans to extend recruitment to include additional sites.

Patient and Public Involvement

There was no direct patient or public involvement groups involved in the study design. The overall study design was developed from previous experience of the investigators involved in the design and coordination of similar studies.

For peer review only

Eligibility Criteria (inclusions/exclusions)

Inclusion criteria

1. Aged between 18 and 75 years (inclusive) at time of screening
2. Aetiology: One or more of:
    a. *Alcohol Related Liver Disease* (No active alcohol misuse ≥6 calendar months prior to screening. Features of chronic liver disease with a compatible history of alcohol excess (>80g/day), in the absence of other causes of chronic liver disease.
    b. *Primary Biliary Cholangitis*
    2 out of: Cholestatic LFTs
    Positive anti-mitochondrial antibody (titre >1:40)
    Compatible Liver Histology
    (If already receiving Ursodeoxycholic Acid must be established on current dose >3 months prior to enrolment)
    c. *Non-Alcoholic Fatty Liver Disease (NAFLD*)
    Either: Histological evidence of hepatic steatosis in the absence of other liver diseases
    Or:
    Imaging compatible with NAFLD (e,g., fatty infiltration of liver) and one or more risk factors

For peer review only

(e.g., elevated BMI, type-2 diabetes mellitus, hypertriglyceridemia, hypertension)

And:

The absence of significant alcohol consumption (<20g/day) and no evidence of other causes of chronic liver disease

d. *Cryptogenic Cirrhosis*

Diagnosis of cirrhosis un-attributable to any other cause

e. *Haemochromatosis*

Diagnosis made on basis of compatible biochemistry (transferrin saturation >60%, ferritin >400), Genotype (homozygous C282Y or H63D compound heterozygote) or histology

f. *Alpha-1 antitrypsin deficiency*

Diagnosis based on compatible genetic, phenotypic or histological testing.

g. *Previous chronic Hepatitis C* (sustained viral response i.e. undetectable HCV RNA 24 weeks after treatment)

3. Diagnosis of cirrhosis – invasive or non-invasive criteria

Cirrhosis defined as Any of:

Biopsy-confirmed diagnosis of cirrhosis

Transient Elastography (TE) - ≥15kPa

Clinical and radiological features which in the opinion of the investigator correlate with a diagnosis of cirrhosis.

For peer review only

4. A MELD Score (Pre-2016) of ≥10 and ≤17 f screening visit

Exclusion criteria

Refusal or inability to give written informed consent to participate in the study.

i) Other causes of chronic liver disease/cirrhosis not included in the listed aetiologies
ii) Portal hypertensive haemorrhage; active episode of bleeding requiring hospitalisation in the last 3 months where varices have not been eradicated by endoscopic band ligation or TIPSS.
iii) Ascites unless, in the opinion of the investigator, is minimal and well controlled with no increase to diuretic therapy in the last 3 months.
iv) Hepatic encephalopathy; current or requiring hospitalisation for treatment in the last 3 months
v) HCC – uncertain cases to be discussed at the local hepatobiliary multidisciplinary team meeting (MDT). Dysplastic or indeterminate nodules to be excluded; regenerative or other nodules to be included at discretion of investigator.
vi) Previous diagnosis of HCC
vii) Previous organ transplant recipient
viii) Listed for liver transplantation
ix) Any situation that in the Investigators opinion may interfere with optimal study participation such as alcohol or drug abuse, domicile too distant from study site, potential non-compliance or inability to co-operate.

For peer review only

x) Presence of clinically relevant acute illness which may preclude on basis of safety.

xi) Presence or history of cancer with exception of adequately treated localised skin carcinoma, in-situ cervical cancer or solid malignancy excised in total, with no recurrence (5-year interval).

xii) Pregnancy or breastfeeding

Interventions

Participants who are randomised to the treatment arm will receive an infusion of the maximum achieved dose up to 1 x 10^9 (day 0). The apheresis product will be collected under the terms of the Human Tissue (Quality and Safety for Human Application) Regulations 2007 No. 1523 enacting the requirements of the EU Tissues and cells Directive (2004/23) and associated Commission Directives at the Apheresis Unit (Royal Infirmary of Edinburgh, Edinburgh, UK). CD14+ monocytes will be isolated, and the macrophage cell product will be manufactured as previously described[24]s, in compliance with GMP regulations under the terms of the SNBTS MIA (IMP) licence at the SNBTS Cell Therapy Facility (Scottish Centre for Regenerative Medicine, Edinburgh, UK).

Each patient will be monitored closely during the infusion to identify potential hypersensitivity reactions and 4-hours post-infusion bloods to monitor for any evidence of macrophage activation syndrome (MAS). A total of 28 participants will be randomised to standard medical care and 28 to receive the cell infusion, allowing for original estimate of 5 drop-outs from each arm. Additional safety data will be collected for the first infusion only for the first three patients randomised to the treatment arm. If it has not been possible to achieve $1x10^9$ macrophages, then the participants will be infused with the quantity obtained, with minimum concentration being 1.25×10^8 cells.

Outcomes

Primary outcome measure

Model of End-Stage Liver Disease (MELD)

The Model for End-stage Liver Disease (MELD) was originally devised to predict survival in patients with complications of portal hypertension undergoing elective placement of trans-jugular intrahepatic portosystemic shunts (TIPSS). The algorithm is based on: creatinine, bilirubin and prothrombin ratio (PTr) and has been demonstrated to be superior to the Child-Turcotte-Pugh (CTP) score in predicting 3-month mortality among patients with end-stage liver disease (ESLD).[25] However, the MELD score has also been applied to predict survival in patients with cirrhosis with infections, variceal haemorrhage, and those with fulminant hepatic failure and alcoholic hepatitis.[26]

Secondary outcome measures

Transplant Free Interval

The number of participants in each of the 2 treatment arms who are transplant free at 12 months will be expressed as proportions and a binomial test will be used for the comparison of proportions between the treatment arm and the control arm. The difference in proportions will be presented along with the 95% confidence interval for the difference in the proportions.

The time to death or transplant will be presented using a Kaplan-Meier survival curve stratified by treatment and accompanied by a log-rank statistic comparing the two arms. Survival estimates with be presented by treatment arm at 3, 6, 9 and 12 months.

Non-Invasive Markers of Fibrosis

Changes in our secondary outcome measures over 90 days up to maximal 360 days as per schedule (Table 1), these include: serum Enhanced Liver Fibrosis (ELF) test (iQur, London, UK, serum Protein Fingerprint™ markers (Nordic Bioscience, Herlev, Denmark), hepatic

For peer review only

Transient Elastography (TE; Echosens, Paris, France) and the United Kingdom Model for End-Stage Liver Disease (UKELD) score.

Enhanced Liver Fibrosis (ELF)

A standardised clinically validated immunoassay test measuring three serum biomarkers which have been shown to correlate to the level of liver fibrosis assessed by liver biopsy, comprising:

- Hyaluronic Acid (HA)
- Tissue Inhibitor of Metalloproteinase 1 (TIMP-1)
- Amino-terminal propeptide of type III procollagen (PIIINP)

The concentrations of each individual protein marker are combined in an algorithm which produces a composite score related to the level of liver fibrosis. The ELF score is a sensitive, specific, and validated method for the non-invasive assessment of hepatic fibrosis in mixed, HCV and NAFLD patient groups.[27]

Protein Fingerprint™ biomarkers

During extracellular matrix (ECM) turnover, proteolytically cleaved matrix degradation fragments, or neoepitopes, are released into the systemic circulation. Cleavage of each ECM protein by specific Matrix Metalloproteinases (MMPs) generates a unique neoepitope. These neoepitopes are more accurate diagnostic and prognostic markers for individual fibroproliferative diseases than their protein of origin. These novel serum biomarkers have been shown to identify patients with progressive fibrosis and permit monitoring of the response to antifibrotic therapy,[28] and also correlate with portal hypertension in patients with cirrhosis.[29]

Transient Elastography (Fibroscan)

For peer review only

Transient elastography is a non-invasive method for assessing liver fibrosis. Mild amplitude and low frequency vibrations (50Hz) are transmitted to the liver tissue, inducing an elastic shear wave that propagates through the underlying liver tissue. The velocity of the wave is directly related to tissue stiffness, considered as a surrogate of the amount of fibrotic tissue. This is expressed as a numerical value in kilopascals (kPa). It is reliable, reproducible with high intra- and inter-observer agreement and has been validated in most causes of chronic liver disease[30]

Chronic Liver Disease Quality of Life questionnaire (CLDQ)

The CLDQ is a liver specific questionnaire for measuring health related quality of life in participants with chronic liver disease. It is self-administered, takes approximately 10 minutes to complete and is designed to reflect the two weeks prior to testing. If necessary, participants can request help to complete this.[31]

It includes 29 items divided into 6 quality of life domains: Abdominal symptoms, Fatigue, Systemic symptoms, Activity, Emotional function and Worry. These items are ranked on a 1 to 7 scale, providing a possible range of scores from 29 (worst quality of life) to 203 (best quality of life). The construct validity of the CLDQ was supported by a strong correlation with participant's global rating scores. It has been shown to be valid and has good test-retest reliability.[32–34]

United Kingdom End Stage Liver Disease (UKELD) score

The UKELD score is readily performed incorporating routine biochemical and haematological indices including bilirubin, albumin, ALT and INR. The UKELD score was developed by the UK Liver Transplant Units to predict transplant waiting list mortality.[35]

The score uses the parameters of Bilirubin (Bil), INR, Creatinine (Creat) and Sodium (Na) in the following algorithm:

For peer review only

*UKELD= [(5.395*ln(INR))+(1.485*ln(Creat)+(3.130*ln(Bil))-(81.565*ln(Na))]+435*

Magnetic resonance imaging and Magnetic Resonance Spectroscopy

Magnetic resonance imaging (MRI) and spectroscopy (MRS) provide methods for the non-invasive assessment of liver microstructure and function. MRI allows for imaging biomarkers to be determined using Liver*Multi*Scan.[36] Tissue microstructure will be investigated using clinically validated metrics. Fibrosis will be assessed by cT_1, iron content with T_2* and the amount of fat in the liver using proton density fat fraction. Organic phosphorus in the liver can be quantified with Phosphorus-31 (^{31}P) MRS[37] a more explorative technique. Using ^{31}P MRS energy metabolism may be investigated via ATP levels and cell membrane integrity by measuring precursors and degradation products. The paired imaging of this study allows for the current utility of MRI to assess disease progression and treatment response to be evaluated

MRI data collected is exploratory and will be according to subgroup analysis: the only planned subgroup analysis is to present the primary outcome for the RCT by disease aetiology (ALD, NAFLD, other). MRI is performed at index visit 2 (or within 7 days) and again at primary outcome timepoint of 90days (+/- 7).

For peer review only

Participant Timeline

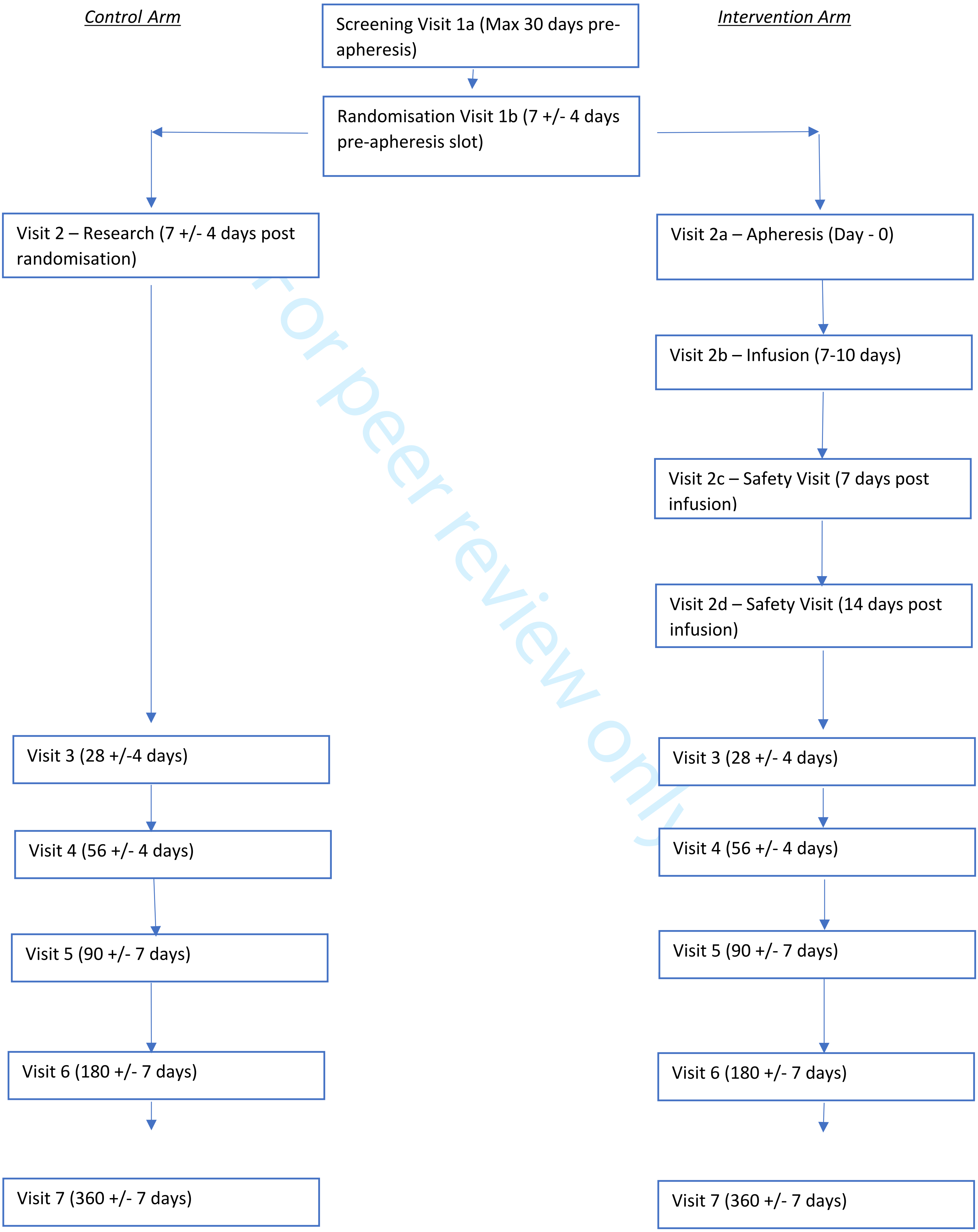

For peer review only

Fig 1: Schematic of Trial Timeline

Sample Size/power calculation

To detect a difference in the baseline to 90-day change in MELD score of 1 standard deviation using a two-sided, two-sample test with a 5% level of significance, a sample size of 23 per group to detect the same level of difference with 90% power is required. All analyses will be carried out on an intention to treat basis, retaining participants in their randomised treatment groups irrespective of the treatment received. Adverse event data will be presented by treatment received.

The number of participants who do not adhere to the protocol is expected to be low. All protocol violations and ineligible participants will be recorded.

Recruitment

Identification of Potential Patients

Potential participants will be identified by their usual direct healthcare team. The treating physician will either introduce the individual to the trial team or ask permission for the trial team to contact them; this could be done through a dedicated invitation letter or a telephone call. The participant information sheet (PIS) will be provided and there will be an opportunity to ask questions. If they agree, a further visit will be scheduled to discuss trial enrolment. This will take place no less than 24 hours later.

For peer review only

Randomisation

Following confirmation of the participant meeting the eligibility criteria, a delegated member of the research team will enter minimal information (participant id, and aetiology) into an online randomisation system, produced for the study by Edinburgh Clinical Trials Unit (ECTU) to determine the treatment allocation. At randomisation, patients will be allocated a unique patient trial number and scheduled for treatment and follow up visits as detailed in the trial schedule.

Allocation

Participants will be assigned to receive either standard medical care or to receive a fresh dose of autologous MDMs at the maximum achievable dose, in a 1:1 ratio based on a minimisation algorithm using the key variable aetiology of disease (ALD, NAFLD, other.) To ensure the allocation is random, participants will be assigned to the group which minimises the imbalance with probability 0.8. If a participant falls into 2 or more strata, then the dominant aetiology (as determined by treating physician) will be used.

Blinding

Due to the nature of the intervention neither participants nor staff can be blinded to allocation of treatment. For some of the additional secondary outcomes we will maintain blinding of external assessors including those processing samples for ELF and protein fingerprint markers. Similarly, there is blinding of MRI physicists and external validation companies responsible for experimental MRI interpretation.

Data Collection

The Case Report Form (CRF) will be completed at set time points as per trial schedule. The CRF will be completed by the Investigator or an authorised member of the research team (as

For peer review only

delegated on the Site Signature and Delegation Log). The exception is the SAE Form which must be signed by the Investigator.

Data reported in each form should be consistent with the source data or the discrepancies should be explained. If information is not known, this must be clearly indicated in the form.

Completed CRFs submitted to the Clinical Research Facility will be reviewed by the Trial Co-ordinator. The data will be entered into an electronic database by designated members of the trial team.

Data Management

The following personal data will be collected as part of the research: Name, date of birth and CHI numbers (Community Health Index; a unique is a 10-character numeric identifier, allocated to each patient on first registration with the NHS system in Scotland). Personal data will be stored in locked cabinets by the research team at the Clinical Research Facilities at each site. Personal data will be stored for 30 years in keeping with the Blood Safety and Quality Regulations. The University of Edinburgh and NHS Lothian are joint data controllers along with any other entities involved in delivering the study that may be a data controller in accordance with applicable laws.

All Investigators and study site staff involved with this study must comply with the requirements of the appropriate data protection legislation (including where applicable the General Data Protection Regulation regarding the collection, storage, processing and disclosure of personal information. Access to personal information will be restricted to individuals from the research team treating the participants, representatives of the sponsor(s) and representatives of regulatory authorities.

For peer review only

Study data will be collected and managed using REDCap electronic data capture tools hosted at The University of Edinburgh. REDCap [38] (Research Electronic Data Capture) is a secure, web-based application designed to support data capture for research studies, providing: an intuitive interface for validated data entry; audit trails for tracking data manipulation and export procedures; automated export procedures for seamless data downloads to common statistical packages; and procedures for importing data from external sources.

Published results will not contain any personal data that could allow identification of individual participants.

Statistical Analysis Plan

The baseline to 90-day change in MELD score will be compared in the two treatment arms using a two-sample t-test or non-parametric equivalent as appropriate. MELD scores calculated for each participant throughout the trial will be used to calculate an area under the curve (AUC) and this will be compared across the groups using a two-sample t-test or non-parametric equivalent as appropriate. In the event of varying durations in the trial follow up, the average AUC per month will be used so that all participants have a comparable measurement.

Changes in secondary outcome measures (ELF score liver stiffness, CLDQ score, transplant-free survival, number of clinical events, UKELD score, blood parameters (bilirubin, albumin, ALT, INR)) over the 1-year study period will be presented graphically by dose. Similarly, these results will used to calculate an AUC for each participant and will be compared across the groups using a two-sample t-test or non-parametric equivalent as appropriate.

The only planned subgroup analysis is to present the primary outcome by disease aetiology (ALD, NAFLD, other). Primary data analysis will be conducted on participants who receive a single infusion versus control; the primary analysis will then be repeated to include those

For peer review only

subjects who receive more than one infusion (3 individuals). There are no plans for an interim analysis.

Data Monitoring

The trial will be coordinated by a Project Management Group, consisting of the grant holders (Chief Investigator and Principal Investigator in Edinburgh), a Trial Manager and coordinating nurse.

The Trial Manager will oversee the study and will be accountable to the Chief Investigator. The Trial Manager, or an authorised member of the research team, will be responsible for checking the CRFs for completeness, plausibility and consistency. Any queries will be resolved by the Investigator or delegated member of the trial team. A Delegation log will be prepared detailing the responsibilities of each member of staff working on the trial.

Safety assessments

The Investigator is responsible for the detection and documentation of events meeting the criteria and definitions detailed within the protocol (available on request). Full details of contraindications and side effects that have been reported following administration of the IMP can be found in the relevant Investigator's Brochure (IB).

Participants will be instructed to contact their Investigator at any time after consenting to join the trial if any symptoms develop. All adverse events (AE) that occur after joining the trial must be reported in detail in the Case Report Form (CRF) or AE form. In the case of an AE, the Investigator should initiate the appropriate treatment according to their medical judgement. Any AE events still present on day 360 will be confirmed and recorded as "ongoing" in the Case Report Form. If appropriate, these should be handed over to the participants' General Practitioner or direct care team.

For peer review only

The ACCORD Research Governance & QA Office is responsible for pharmacovigilance reporting on behalf of the co-sponsors (University of Edinburgh and NHS Lothian).

The ACCORD Research Governance & QA Office has a legal responsibility to notify the regulatory competent authority and relevant ethics committee (Research Ethics Committee (REC) that approved the trial). Fatal or life threatening SUSARs will be reported no later than 7 calendar days and all other SUSARs will be reported no later than 15 calendar days after ACCORD is first aware of the reaction.

ACCORD will inform Investigators at participating sites of all SUSARs and any other arising safety information.

An Annual Safety Report/Development Safety Update Report will be submitted, by ACCORD, to the regulatory authorities and RECs listing all SARs and SUSARs.

Monitoring and Oversight

An ACCORD Clinical Trials Monitor, or an appointed monitor will visit the Investigator site prior to the start of the study and during the course of the study if required, in accordance with the monitoring plan if required. Risk assessment will determine if audit, by the ACCORD QA group, is required. Details will be captured in an audit plan.

Discussion

MATCH is a randomised controlled trial designed to identify whether there is a measurable improvements in MELD and relevant secondary fibrosis assessments following autologous macrophage therapy. It builds upon the safety and feasibility assessment of the earlier phase I trial. Through this trial, we aim to add to the collective knowledge of this potential new therapeutic modality for liver disease in this patient population who currently have limited treatment options. If effective, autologous macrophage cell therapy could improve clinical outcomes and enhance HRQoL in people with cirrhosis.

For peer review only

Following initial trial results, we expect that a further extended study will be necessary to determine longer term safety and the durability of treatment responses. Moreover, it is not yet clear whether patients may require repeat treatments to maximise efficacy.

We hope that this initial phase II trial will provide robust evidence to support and inform future trial design.

For peer review only

Contributors: FM and SJF was responsible for the conceptualisation and design of the trial. PNB is study clinician and drafted manuscript and provided critical review of protocol. JAF aided manuscript preparation and critical appraisal. CG was responsible for statistical design. AG, CP, NWAM, ARF, MLT and JDMC were responsible reviewing sections around product manufacture. MM and TM provided manuscript review and critique. SIKS and DMM developed section on MRI imaging. NL and JFD provided critical appraisal of manuscript. All authors critically revised and approved the manuscript.

Conflict of Interests: PNB has received honoraria from Takeda. JAF has received consultancy fees for Ferring Pharmaceuticals, Macrophage Pharma, Aquilla BioMedical, Caldan Therapeutics, Cypralis Ltd, Third Rock Ventures, Rallybio, Narrow River Management, Gilde Healthcare, Guidepoint, Techspert.io and acted as advisory board member for: Novartis, Galecto Biotech, Tectonic Therapeutic and received research grant funding from Novartis and Intercept Pharmaceuticals. JFD has received honoraria and research grants from Gilead, AbbVie and MSD. JDMC and SJF are founders and scientific advisers to Resolution Therapeutics Ltd.

For peer review only

Bibliography

1. Asrani, S. K., Devarbhavi, H., Eaton, J. & Kamath, P. S. Burden of liver diseases in the world. *Journal of Hepatology* vol. 70 151–171 (2019).

2. Pimpin, L. *et al.* Burden of liver disease in Europe: Epidemiology and analysis of risk factors to identify prevention policies. *Journal of Hepatology* vol. 69 718–735 (2018).

3. Sepanlou, S. G. *et al.* The global, regional, and national burden of cirrhosis by cause in 195 countries and territories, 1990–2017: a systematic analysis for the Global Burden of Disease Study 2017. *The Lancet Gastroenterology and Hepatology* **5**, 245–266 (2020).

4. Younossi, Z. & Henry, L. Overall health-related quality of life in patients with end-stage liver disease. *Clinical Liver Disease* vol. 6 9–14 (2015).

5. Van Der Plas, S. M. *et al.* Generic and disease-specific health related quality of life of liver patients with various aetiologies: A survey. *Quality of Life Research* **16**, 375–388 (2007).

6. D'Amico, G., Garcia-Tsao, G. & Pagliaro, L. Natural history and prognostic indicators of survival in cirrhosis: A systematic review of 118 studies. *Journal of Hepatology* vol. 44 217–231 (2006).

7. Ratib, S., Fleming, K. M., Crooks, C. J., Aithal, G. P. & West, J. 1 and 5 year survival estimates for people with cirrhosis of the liver in England, 1998-2009: A large population study. *Journal of Hepatology* **60**, 282–289 (2014).

8. Arroyo, V. *et al.* The systemic inflammation hypothesis: Towards a new paradigm of acute decompensation and multiorgan failure in cirrhosis. *Journal of Hepatology* vol. 74 670–685 (2021).

9. Fallowfield, J. A., Jimenez-Ramos, M. & Robertson, A. Emerging synthetic drugs for the treatment of liver cirrhosis. *Expert Opinion on Emerging Drugs* 1–16 (2021) doi:10.1080/14728214.2021.1918099.

10. Neuberger, J. Liver transplantation in the United Kingdom. *Liver Transplantation* **22**, 1129–1135 (2016).

11. Ransford, R., Gunson, B., Mayer, D., Neuberger, J. & Christensen, E. Effect on outcome of the lengthening waiting list for liver transplantation. *Gut* vol. 47 441–443 (2000).

12. Moini, M., Schilsky, M. L. & Tichy, E. M. Review on immunosuppression in liver transplantation. *World Journal of Hepatology* vol. 7 1355–1368 (2015).

13. Forbes, S. J., Gupta, S. & Dhawan, A. Cell therapy for liver disease: From liver transplantation to cell factory. *Journal of Hepatology* vol. 62 S157–S169 (2015).

14. Dwyer, B. J., Macmillan, M. T., Brennan, P. N. & Forbes, S. J. Cell therapy for advanced liver diseases: Repair or rebuild. *Journal of Hepatology* (2020) doi:10.1016/j.jhep.2020.09.014.

15. Spahr, L. *et al.* Autologous Bone Marrow Mononuclear Cell Transplantation in Patients with Decompensated Alcoholic Liver Disease: A Randomized Controlled Trial. *PLoS ONE* **8**, (2013).

16. Newsome, P. N. *et al.* Granulocyte colony-stimulating factor and autologous CD133-positive stem-cell therapy in liver cirrhosis (REALISTIC): an open-label, randomised, controlled phase 2 trial. *The Lancet Gastroenterology and Hepatology* **3**, 25–36 (2018).

For peer review only

17. Tacke, F. Functional role of intrahepatic monocyte subsets for the progression of liver inflammation and liver fibrosis in vivo. *Fibrogenesis & Tissue Repair* **5**, S27 (2012).

18. Tacke, F. & Zimmermann, H. W. Macrophage heterogeneity in liver injury and fibrosis. *Journal of Hepatology* vol. 60 1090–1096 (2014).

19. Thomas, J. A. *et al.* Macrophage therapy for murine liver fibrosis recruits host effector cells improving fibrosis, regeneration, and function. *Hepatology* **53**, 2003–2015 (2011).

20. Ramachandran, P. *et al.* Differential Ly-6C expression identifies the recruited macrophage phenotype, which orchestrates the regression of murine liver fibrosis. *Proceedings of the National Academy of Sciences of the United States of America* **109**, E3186–E3195 (2012).

21. Bird, T. G. *et al.* Bone marrow injection stimulates hepatic ductular reactions in the absence of injury via macrophage-mediated TWEAK signaling. *Proceedings of the National Academy of Sciences of the United States of America* **110**, 6542–6547 (2013).

22. Moore, J. K. *et al.* Phenotypic and functional characterization of macrophages with therapeutic potential generated from human cirrhotic monocytes in a cohort study. *Cytotherapy* **17**, 1604–1616 (2015).

23. Moroni, F. *et al.* Safety profile of autologous macrophage therapy for liver cirrhosis. *Nature Medicine* **25**, 1560–1565 (2019).

24. Fraser, A. R. *et al.* Development, functional characterization and validation of methodology for GMP-compliant manufacture of phagocytic macrophages: A novel cellular therapeutic for liver cirrhosis. *Cytotherapy* **19**, 1113–1124 (2017).

25. Cuomo, O., Perrella, A. & Arenga, G. Model for End-Stage Liver Disease (MELD) Score System to Evaluate Patients With Viral Hepatitis on the Waiting List: Better Than the Child-Turcotte-Pugh (CTP) System? *Transplantation Proceedings* **40**, 1906–1909 (2008).

26. Kamath, P. S. & Kim, W. R. The Model for End-stage Liver Disease (MELD). *Hepatology* vol. 45 797–805 (2007).

27. Rosenberg, W. M. C. *et al.* Serum markers detect the presence of liver fibrosis: A cohort study. *Gastroenterology* **127**, 1704–1713 (2004).

28. Leeming, D. J. *et al.* Pro-C5, a marker of true type V collagen formation and fibrillation, correlates with portal hypertension in patients with alcoholic cirrhosis. *Scandinavian Journal of Gastroenterology* **50**, 584–592 (2015).

29. Karsdal, M. A. *et al.* Review article: The efficacy of biomarkers in chronic fibroproliferative diseases - Early diagnosis and prognosis, with liver fibrosis as an exemplar. *Alimentary Pharmacology and Therapeutics* vol. 40 233–249 (2014).

30. Fraquelli, M. *et al.* Reproducibility of transient elastography in the evaluation of liver fibrosis in patients with chronic liver disease. *Gut* **56**, 968–973 (2007).

31. Younossi, Z. M., Guyatt, G., Kiwi, M., Boparai, N. & King, D. Development of a disease specific questionnaire to measure health related quality of life in patients with chronic liver disease. *Gut* **45**, 295–300 (1999).

For peer review only

32. Lam, E. T. P., Lam, C. L. K., Lai, C. L., Yuen, M. F. & Fong, D. Y. T. Psychometrics of the chronic liver disease questionnaire for Southern Chinese patients with chronic hepatitis B virus infection. *World Journal of Gastroenterology* **15**, 3288–3297 (2009).

33. Kallman, J. *et al.* Fatigue and health-related quality of life (HRQL) in chronic hepatitis C virus infection. *Digestive Diseases and Sciences* **52**, 2531–2539 (2007).

34. Chawla, K. S. *et al.* Reliability and validity of the Chronic Liver Disease Questionnaire (CLDQ) in adults with non-alcoholic steatohepatitis (NASH). *BMJ Open Gastroenterology* **3**, (2016).

35. Barber, K. *et al.* Elective liver transplant list mortality: Development of a United Kingdom end-stage liver disease score. *Transplantation* **92**, 469–476 (2011).

36. Pavlides, M. *et al.* Multiparametric magnetic resonance imaging predicts clinical outcomes in patients with chronic liver disease. *Journal of Hepatology* **64**, 308–315 (2016).

37. Valkovič, L., Chmelík, M. & Krššák, M. In-vivo 31P-MRS of skeletal muscle and liver: A way for non-invasive assessment of their metabolism. *Analytical Biochemistry* **529**, 193–215 (2017).

38. Harris, P. A. *et al.* Research electronic data capture (REDCap)-A metadata-driven methodology and workflow process for providing translational research informatics support. *Journal of Biomedical Informatics* **42**, 377–381 (2009).

For peer review only

			Treatment group				Control group					
	**Screening**	**Randomisation**	**Apheresis**	**Cell Infusion**	**Safety Visit**	**Safety Visit**	**Research sample**	**Follow-up**	**Follow-up**	**Follow-up**	**Follow-up**	**Follow-up**
	**Visit 1a**	**Visit 1b**	**Visit 2a Within 7±4 days of Visit 1b**	**Visit 2b 7>10days after apheresis (Day 0)**	**Visit 2c (Day 7)**	**Visit 2d (Day 14)**	**Visit 2 (day 7±4days from visit 1b)**	**Visit 3 (Day 28±4 days)**	**Visit 4 (Day 56±4 days)**	**Visit 5 (Day 90±7 days)**	**Visit 6 (Day 180±7 days)**	**Visit 7 (Day 360±7 days)**
Informed consent	X											
Clinical Assessment	X	X	X	X	X	X	X	X	X	X	X	X
Vital Signs	X	X	X	X	X	X		X	X	X	X	X
Screening Blood Tests	X											
ECG	X											
Standard Blood Tests	X	X		X	X	X		X	X	X	X	X
Research Bloods* ***			X		X	X	X	X	X	X	X	X
Mandatory Microbiology	X		X									
Ferritin	X			X##	X	X						
Triglyceride	X			X##	X	X						
Pre-infusion blood tests				X								
MELD/UKELD	X	X		X	X	X		X	X	X	X	X
Pregnancy test	X*	X*		X*	X**							X*
Abdominal USS	X[1]										X[1]	X[1]
Fibroscan	X									X	X	X
ELF Panel	X							X	X	X	X	X
Protein Fingerprint™	X[1]							X[1]		X[1]		
CLDQ		X								X	X	X
31P MRS MRI#	X***									X		
Adverse Events	X	X	X	X	X	X	X	X	X	X	X	X
Clinical Events	X	X	X	X	X	X	X	X	X	X	X	X
Concomitant Medication	X	X	X	X	X	X	X	X	X	X	X	X

*****women of child bearing age only *** If test not carried out at previous visit* **** If pass screen & before visit 2b [1]fasted visit #RIE patients only ##obtain before discharge*

Table 1: Trial Assessment Schedule

Reporting checklist for protocol of a clinical trial.

Based on the SPIRIT guidelines.

Instructions to authors

Complete this checklist by entering the page numbers from your manuscript where readers will find each of the items listed below.

Your article may not currently address all the items on the checklist. Please modify your text to include the missing information. If you are certain that an item does not apply, please write "n/a" and provide a short explanation.

Upload your completed checklist as an extra file when you submit to a journal.

In your methods section, say that you used the SPIRITreporting guidelines, and cite them as:

Chan A-W, Tetzlaff JM, Gøtzsche PC, Altman DG, Mann H, Berlin J, Dickersin K, Hróbjartsson A, Schulz KF, Parulekar WR, Krleža-Jerić K, Laupacis A, Moher D. SPIRIT 2013 Explanation and Elaboration: Guidance for protocols of clinical trials. BMJ. 2013;346:e7586

For peer review only

		Reporting Item	Page Number
**Administrative information**			
Title	#1	Descriptive title identifying the study design, population, interventions, and, if applicable, trial acronym	1
Trial registration	#2a	Trial identifier and registry name. If not yet registered,	2

		name of intended registry	
Trial registration: data set	#2b	All items from the World Health Organization Trial Registration Data Set	N/A
Protocol version	#3	Date and version identifier	3
Funding	#4	Sources and types of financial, material, and other support	3
Roles and responsibilities: contributorship	#5a	Names, affiliations, and roles of protocol contributors	26
Roles and responsibilities: sponsor contact information	#5b	Name and contact information for the trial sponsor	9
Roles and responsibilities: sponsor and funder	#5c	Role of study sponsor and funders, if any, in study design; collection, management, analysis, and interpretation of data; writing of the report; and the decision to submit the report for publication, including whether they will have ultimate authority over any of these activities	9
Roles and responsibilities: committees	#5d	Composition, roles, and responsibilities of the coordinating centre, steering committee, endpoint adjudication committee, data management team, and other individuals or groups overseeing the trial, if applicable (see Item 21a for data monitoring committee)	9

Introduction

For peer review only

Background and rationale	#6a	Description of research question and justification for undertaking the trial, including summary of relevant studies (published and unpublished) examining benefits and harms for each intervention	5-8
Background and rationale: choice of comparators	#6b	Explanation for choice of comparators	N/A
Objectives	#7	Specific objectives or hypotheses	9
Trial design	#8	Description of trial design including type of trial (eg, parallel group, crossover, factorial, single group), allocation ratio, and framework (eg, superiority, equivalence, non-inferiority, exploratory)	7,19
**Methods: Participants, interventions, and outcomes**			
Study setting	#9	Description of study settings (eg, community clinic, academic hospital) and list of countries where data will be collected. Reference to where list of study sites can be obtained	9, 19
Eligibility criteria	#10	Inclusion and exclusion criteria for participants. If applicable, eligibility criteria for study centres and individuals who will perform the interventions (eg,	10, 11, 12

For peer review only

		surgeons, psychotherapists)	
Interventions: description	#11a	Interventions for each group with sufficient detail to allow replication, including how and when they will be administered	12
Interventions: modifications	#11b	Criteria for discontinuing or modifying allocated interventions for a given trial participant (eg, drug dose change in response to harms, participant request, or improving / worsening disease)	N/A
Interventions: adherance	#11c	Strategies to improve adherence to intervention protocols, and any procedures for monitoring adherence (eg, drug tablet return; laboratory tests)	30
Interventions: concomitant care	#11d	Relevant concomitant care and interventions that are permitted or prohibited during the trial	N/A
Outcomes	#12	Primary, secondary, and other outcomes, including the specific measurement variable (eg, systolic blood pressure), analysis metric (eg, change from baseline, final value, time to event), method of aggregation (eg, median, proportion), and time point for each outcome. Explanation of the clinical relevance of chosen efficacy and harm outcomes is strongly recommended	14-17
Participant timeline	#13	Time schedule of enrolment, interventions (including any run-ins and washouts), assessments, and visits for participants. A schematic diagram is highly recommended (see Figure)	18

For peer review only

Sample size	#14	Estimated number of participants needed to achieve study objectives and how it was determined, including clinical and statistical assumptions supporting any sample size calculations	19
Recruitment	#15	Strategies for achieving adequate participant enrolment to reach target sample size	1920
**Methods: Assignment of interventions (for controlled trials)**			
Allocation: sequence generation	#16a	Method of generating the allocation sequence (eg, computer-generated random numbers), and list of any factors for stratification. To reduce predictability of a random sequence, details of any planned restriction (eg, blocking) should be provided in a separate document that is unavailable to those who enrol participants or assign interventions	20
Allocation concealment mechanism	#16b	Mechanism of implementing the allocation sequence (eg, central telephone; sequentially numbered, opaque, sealed envelopes), describing any steps to conceal the sequence until interventions are assigned	20
Allocation: implementation	#16c	Who will generate the allocation sequence, who will enrol participants, and who will assign participants to interventions	20

For peer review only

Blinding (masking)	#17a	Who will be blinded after assignment to interventions (eg, trial participants, care providers, outcome assessors, data analysts), and how	20
Blinding (masking): emergency unblinding	#17b	If blinded, circumstances under which unblinding is permissible, and procedure for revealing a participant's allocated intervention during the trial	N/A
**Methods: Data collection, management, and analysis**			
Data collection plan	#18a	Plans for assessment and collection of outcome, baseline, and other trial data, including any related processes to promote data quality (eg, duplicate measurements, training of assessors) and a description of study instruments (eg, questionnaires, laboratory tests) along with their reliability and validity, if known. Reference to where data collection forms can be found, if not in the protocol	20, 21
Data collection plan: retention	#18b	Plans to promote participant retention and complete follow-up, including list of any outcome data to be collected for participants who discontinue or deviate from intervention protocols	20, 21
Data management	#19	Plans for data entry, coding, security, and storage, including any related processes to promote data quality (eg, double data entry; range checks for data values).	21

For peer review only

		Reference to where details of data management procedures can be found, if not in the protocol	
Statistics: outcomes	#20a	Statistical methods for analysing primary and secondary outcomes. Reference to where other details of the statistical analysis plan can be found, if not in the protocol	22
Statistics: additional analyses	#20b	Methods for any additional analyses (eg, subgroup and adjusted analyses)	22
Statistics: analysis population and missing data	#20c	Definition of analysis population relating to protocol non-adherence (eg, as randomised analysis), and any statistical methods to handle missing data (eg, multiple imputation)	N/A
**Methods: Monitoring**			
Data monitoring: formal committee	#21a	Composition of data monitoring committee (DMC); summary of its role and reporting structure; statement of whether it is independent from the sponsor and competing interests; and reference to where further details about its charter can be found, if not in the protocol. Alternatively, an explanation of why a DMC is not needed	9
Data monitoring: interim analysis	#21b	Description of any interim analyses and stopping guidelines, including who will have access to these interim results and make the final decision to terminate the trial	9, 23
Harms	#22	Plans for collecting, assessing, reporting, and managing solicited and spontaneously reported adverse events and other unintended effects of trial interventions or trial	20, 21, 23

For peer review only

		conduct	
Auditing	#23	Frequency and procedures for auditing trial conduct, if any, and whether the process will be independent from investigators and the sponsor	23
**Ethics and dissemination**			
Research ethics approval	#24	Plans for seeking research ethics committee / institutional review board (REC / IRB) approval	2, 9
Protocol amendments	#25	Plans for communicating important protocol modifications (eg, changes to eligibility criteria, outcomes, analyses) to relevant parties (eg, investigators, REC / IRBs, trial participants, trial registries, journals, regulators)	N/A
Consent or assent	#26a	Who will obtain informed consent or assent from potential trial participants or authorised surrogates, and how (see Item 32)	9, 12, 23
Consent or assent: ancillary studies	#26b	Additional consent provisions for collection and use of participant data and biological specimens in ancillary studies, if applicable	N/A
Confidentiality	#27	How personal information about potential and enrolled participants will be collected, shared, and maintained in order to protect confidentiality before, during, and after the trial	20, 21
Declaration of	#28	Financial and other competing interests for principal	25

For peer review only

interests		investigators for the overall trial and each study site	
Data access	#29	Statement of who will have access to the final trial dataset, and disclosure of contractual agreements that limit such access for investigators	20, 21, 24
Ancillary and post trial care	#30	Provisions, if any, for ancillary and post-trial care, and for compensation to those who suffer harm from trial participation	N/A
Dissemination policy: trial results	#31a	Plans for investigators and sponsor to communicate trial results to participants, healthcare professionals, the public, and other relevant groups (eg, via publication, reporting in results databases, or other data sharing arrangements), including any publication restrictions	2
Dissemination policy: authorship	#31b	Authorship eligibility guidelines and any intended use of professional writers	N/A
Dissemination policy: reproducible research	#31c	Plans, if any, for granting public access to the full protocol, participant-level dataset, and statistical code	N/A
**Appendices**			
Informed consent materials	#32	Model consent form and other related documentation given to participants and authorised surrogates	N/A
Biological specimens	#33	Plans for collection, laboratory evaluation, and storage of biological specimens for genetic or molecular analysis in the current trial and for future use in ancillary studies, if applicable	N/A

For peer review only

The SPIRIT Explanation and Elaboration paper is distributed under the terms of the Creative Commons Attribution License CC-BY-NC. This checklist was completed on 05. May 2021 using https://www.goodreports.org/, a tool made by the EQUATOR Network in collaboration with Penelope.ai

For peer review only

BMJ Open

Study Protocol: A multicentre, open-label, parallel-group, phase 2, randomised controlled trial of autologous macrophage therapy for liver cirrhosis (MATCH)

Journal:	*BMJ Open*
Manuscript ID	bmjopen-2021-053190.R1
Article Type:	Protocol
Date Submitted by the Author:	26-Sep-2021

Complete List of Authors:	Brennan, Paul; The University of Edinburgh Edinburgh Medical School, Centre for Regenerative Medicine<br>MacMillan, Mark; The University of Edinburgh Edinburgh Medical School<br>Manship, Thomas; NHS Lothian, CLDD<br>Moroni, Francesca; NHS Grampian<br>Glover, Alison; Scottish National Blood Transfusion Service<br>Graham, Catriona; The University of Edinburgh<br>Semple, Scott; The University of Edinburgh Deanery of Clinical Sciences, Centre for Cardiovascular Science<br>Morris, David; The University of Edinburgh Deanery of Clinical Sciences, Centre for Cardiovascular Science<br>Fraser, Alasdair; SNBTS, Tissues, Cells and Advanced Therapeutics<br>Pass, Chloe; SNBTS, Tissues, Cells and Advanced Therapeutics<br>McGowan, Neil; SNBTS, Tissues, Cells and Advanced Therapeutics<br>Turner, Marc; SNBTS, Tissues, Cells and Advanced Therapeutics<br>Lachlan, Neil; NHS Greater Glasgow and Clyde<br>Dillon, John; University of Dundee Division of Cardiovascular and Diabetes Medicine, Liver Group<br>Campbell, John; SNBTS, Tissues, Cells and Advanced Therapeutics<br>Fallowfield, Jonathan; University of Edinburgh MRC Centre for Inflammation Research, Queen's Medical Research Institute<br>Forbes, Stuart; The University of Edinburgh Deanery of Clinical Sciences, Centre for Regenerative Medicine

**Primary Subject Heading**:	Gastroenterology and hepatology
Secondary Subject Heading:	Research methods, Pharmacology and therapeutics
Keywords:	Hepatobiliary disease < GASTROENTEROLOGY, Immunology < NATURAL SCIENCE DISCIPLINES, Clinical trials < THERAPEUTICS, Cell biology < NATURAL SCIENCE DISCIPLINES

SCHOLARONE™ Manuscripts

I, the Submitting Author has the right to grant and does grant on behalf of all authors of the Work (as defined in the below author licence), an exclusive licence and/or a non-exclusive licence for contributions from authors who are: i) UK Crown employees; ii) where BMJ has agreed a CC-BY licence shall apply, and/or iii) in accordance with the terms applicable for US Federal Government officers or employees acting as part of their official duties; on a worldwide, perpetual, irrevocable, royalty-free basis to BMJ Publishing Group Ltd ("BMJ") its licensees and where the relevant Journal is co-owned by BMJ to the co-owners of the Journal, to publish the Work in this journal and any other BMJ products and to exploit all rights, as set out in our licence.

The Submitting Author accepts and understands that any supply made under these terms is made by BMJ to the Submitting Author unless you are acting as an employee on behalf of your employer or a postgraduate student of an affiliated institution which is paying any applicable article publishing charge ("APC") for Open Access articles. Where the Submitting Author wishes to make the Work available on an Open Access basis (and intends to pay the relevant APC), the terms of reuse of such Open Access shall be governed by a Creative Commons licence – details of these licences and which Creative Commons licence will apply to this Work are set out in our licence referred to above.

Other than as permitted in any relevant BMJ Author's Self Archiving Policies, I confirm this Work has not been accepted for publication elsewhere, is not being considered for publication elsewhere and does not duplicate material already published. I confirm all authors consent to publication of this Work and authorise the granting of this licence.

For peer review only

Study Protocol: A multicentre, open-label, parallel-group, phase 2, randomised controlled trial of autologous macrophage therapy for liver cirrhosis (MATCH)

Paul N Brennan[1,3*], Mark MacMillan[1], Thomas Manship[3], Francesca Moroni[5], Alison Glover[2], Catriona Graham[4], Scott IK Semple[6], David M Morris[6], Alasdair R Fraser[2], Chloe Pass[2], Neil WA McGowan[2], Marc L Turner[2], Neil Lachlan[7], John F Dillon[8], John DM Campbell[2], Jonathan A Fallowfield[3,9], Stuart J Forbes[1,3]

1. Centre for Regenerative Medicine, University of Edinburgh, Edinburgh, UK
2. Scottish National Blood Transfusion Service (SNBTS), Edinburgh, UK
3. Centre for Liver and Digestive Disorders, Royal Infirmary of Edinburgh, Edinburgh, UK
4. Wellcome Trust Clinical Research Facility, University of Edinburgh, Edinburgh, UK
5. Aberdeen Royal Infirmary, NHS Grampian, Foresterhill Health Campus, Aberdeen, UK
6. The University of Edinburgh Deanery of Clinical Sciences, Centre for Cardiovascular Science, Edinburgh, UK
7. NHS Greater Glasgow and Clyde, Glasgow Royal Infirmary, Glasgow, UK
8. University of Dundee, Ninewells Hospital and Medical School, Dundee, UK
9. Centre for Inflammation Research, University of Edinburgh, Edinburgh, UK

*Corresponding author: Pbrenna2@ed.ac.uk

Word Count: 5,523 (Excl. References and Tables)

For peer review only

Abstract

Introduction

Liver cirrhosis is a growing global healthcare challenge. Cirrhosis is characterized by severe liver fibrosis, organ dysfunction and complications related to portal hypertension. There are no licensed antifibrotic or pro-regenerative medicines and liver transplantation is a scarce resource. Hepatic macrophages can promote both liver fibrogenesis and fibrosis regression. The safety and feasibility of peripheral infusion of ex vivo matured autologous monocyte-derived macrophages in patients with compensated cirrhosis has been demonstrated.

Methods and Analysis

The efficacy of autologous macrophage therapy, compared to standard medical care, will be investigated in a cohort of adult patients with compensated cirrhosis in a multicentre, open-label, parallel-group, phase 2, randomised controlled trial. The primary outcome is the change in Model for End-Stage Liver Disease (MELD) score at 90 days. The trial will provide the first high-quality examination of the efficacy of autologous macrophage therapy in improving liver function, non-invasive fibrosis markers and other clinical outcomes in patients with compensated cirrhosis.

Ethics and dissemination

The trial will be conducted according to the ethical principles of the Declaration of Helsinki 2013 and has been approved by Scotland A Research Ethics Committee (reference 15/SS/0121), NHS Lothian Research and Development department and the Medicine and Health Care Regulatory Agency (MHRA-UK). Final results will be presented in peer-reviewed journals and at relevant conferences.

Trial registration

The trial was registered prospectively in the International Standard Randomized Controlled Trial Number (ISRCTN) Registry (ISRCTN10368050) and European Union Clinical Trials Register (EudraCT; reference 2015-000963-15).

For peer review only

Protocol V14 – July 2020

Funding Statement

This work was supported by a Medical Research Council UK grant (Biomedical Catalyst Major Awards Committee, Reference: MR/M007588/1) to Prof. Stuart Forbes

Keywords: *Liver cirrhosis, macrophages, cell therapy, liver fibrosis, liver regeneration*

For peer review only

Strengths and limitations of this study

- First randomised controlled trial of an innovative cell-based therapy for cirrhosis
- Range of evidence-based non-invasive assessments of liver fibrosis and function
- Concurrent longitudinal measurement of health-related quality of life in an important chronic liver disease population
- Open label design, but outcome assessors blinded to treatment allocation

For peer review only

Introduction

Liver disease is responsible for almost 2 million deaths per year globally, 1 million directly relating to complications of end-stage liver failure (ESLF) and a further 1 million due complications of hepatitis including hepatocellular carcinoma (HCC).[1] Cirrhosis and liver cancer are now respectively the 11th and 16th most common cause of death globally, accounting for 3.5% of all deaths. Variation in liver disease epidemiology occurs relative to the prevalence of modifiable risk factors including harmful alcohol ingestion, obesity/metabolic syndrome and viral hepatitis.[2] Worldwide there were 10·6 million prevalent cases of decompensated cirrhosis and 112 million prevalent cases of compensated cirrhosis in 2017.[3]

Cirrhosis represents the end-stage of chronic liver injury and progressive fibrosis (scarring), irrespective of the underlying aetiology. It is characterised by severe liver fibrosis leading to architectural disruption, hepatocyte dysfunction and portal hypertension. Cirrhosis typically affects those of working age, which has broad socio-economic impacts. Furthermore, cirrhosis impairs health-related quality of life (HRQoL) including mental health and physical factors and reduced ability to perform activities of daily living[4]; those with primary biliary cholangitis (PBC), non-alcoholic fatty liver disease (NAFLD) and hepatitis C virus (HCV) appear disproportionately affected.[5]

The classical dichotomy of chronic liver disease staging is compensated (asymptomatic) or decompensated cirrhosis. Acute decompensation delineates the development of one or more associated sequelae and is a key prognostic inflection point. The transition from compensated to decompensated cirrhosis occurs at a rate of about 5-7% per year.[6] Decompensation represents a prognostic milestone as it significantly alters mortality, with a cumulative 1 year mortality of 77% for those with stage 3 and 4 decompensated disease vs 4.4% in those with compensated disease. Importantly, emergency hospitalisation for decompensated liver disease heralds a deterioration in a patient's prognosis independent of stage of cirrhosis.[7]

Cirrhosis decompensation heralds the development of widespread organ dysregulation, including portal hypertension, splanchnic vasodilation, left ventricular impairment and systemic immune dysfunction. Inflammatory mediators of liver disease may underpin and potentiate nitric oxide-mediated capillary dysfunction, direct immunocytopathy and induce significant metabolic derangement, and redistribution of essential nutrient precursors.[8]

For patients in whom disease-specific therapy is unsuccessful or not possible, treatment options remain limited. Presently, although numerous agents have been evaluated in clinical trials, there are no approved pharmacological therapies for reversing fibrosis or stimulating liver regeneration in the cirrhotic liver.[9] Liver transplantation remains the only curative option for those with end-stage cirrhosis or HCC. Unfortunately, a significant proportion of those referred for transplant assessment are ineligible and ~12% die annually while on the waiting list in the UK.[10,11] Those who do undergo liver transplantation require lifelong immunosuppression with inherent risks of toxicity and adverse effects.[12]

Although whole organ or split liver transplantation are well established procedures to reinstate liver functional capacity, cell-based transplantation approaches are emerging.[13] Successful cell therapy could theoretically overcome organ availability limitations, whilst avoiding invasive surgical interventions. Successful hepatocyte transplantation involves reconstitution of as little as 1- 2.5% of functional tissue across a range of inherited metabolic liver diseases and highlights the utility of such approaches.[14] Furthermore, there is a requirement for treatments that can 'bridge' patients with cirrhosis until a donor organ is available or allow spontaneous regeneration to occur following acute liver failure (ALF). Cell therapies that sufficiently modulate cirrhosis by reducing fibrosis and stimulating liver function may also promote endogenous tissue repair and regeneration such that the need for transplantation is delayed or obviated.

For peer review only

Previous studies have typically focussed on the use of mesenchymal stem cells (MSCs), Hepatocyte Stem Cells (HSCs) and heterogenous cell populations which will include pro-inflammatory and pro-fibrotic cell lineages. Despite promising preclinical studies, randomised controlled trials of autologous cell therapies in cirrhosis have so far been disappointing.[15,16]

Macrophages are a heterogeneous, highly plastic population of cells with a diverse spectrum of roles within the liver including phagocytosis and maintenance of immune tolerance. Hepatic monocyte-derived macrophages are known to play a dual role in liver fibrosis. During chronic liver injury models they mediate the recruitment of pro-inflammatory cells and activation of hepatic stellate cells to promote fibrogenesis.[14] Conversely, fibrosis regression is characterised by an in situ phenotypic switch to a restorative hepatic macrophage population with pro-resolution properties[17] whereby liver repair and regeneration is facilitated by increased expression of matrix metalloproteinases (MMPs), growth factors, and phagocytosis-related genes.[18,19] This process of phenotypic "switching" from a pro-inflammatory "M1-like" moiety, to a pro-resolution "M2-like" macrophage is mediated via down-regulation of NOD-, LRR- and pyrin domain-containing protein 3 (NLRP3).[14]

In a mouse model of chronic liver injury, cell therapy with unmanipulated syngenic macrophages reduced fibrosis and improved markers of liver function.[20] Furthermore, infusion of human macrophages (differentiated from cirrhotic patients' apheresis-derived $CD14^+$ monocytes) also resolved liver fibrosis in mice, indicating their suitability for clinical therapy.[21–23]

We recently demonstrated the feasibility of performing apheresis in cirrhotic patients and differentiating autologous bone marrow derived monocytes into macrophages.[24] This process includes specific $CD14^+$ monocyte isolation from peripheral circulation leucopharesis collections using CliniMACS automated separation device, a closed-system, where the product is incubated with CD14 labelled magnetic beads, allowing separation of $CD14^+$ cells

For peer review only

when passed over a magnetic column. Selected $CD14^+$ monocytes are counted and re-suspended in differentiation medium containing 100ng/mL Macrophage Colony-Stimulating Factor (M-CSF). Cells are placed into closed-system, low adhesion culture bags at optimum cell density ($2x10^6$ cells per mL and per cm^3). Cells are cultured in a humidified atmosphere at 37°C, with 5% CO2, for 7 days. Media replenishment is undertaken twice during culture (typically days 3 and 5), using differentiation media supplemented with 100ng/mL M-CSF. Flow cytometry is used to determine cell viability and phenotype cell populations pre- and post-monocyte selection and post-macrophage differentiation prior to product release, this has been validated for 7 and 10-day timepoints.

We also have extensive pre-clinical data demonstrating that peripherally injected macrophages hone to the liver (predominantly) and spleen (after passing rapidly through the lungs) and that this process in enhanced in the presence of liver damage.[20,25] Furthermore, in a first-in-human study we confirmed the safety, feasibility and maximum achievable dose of autologous macrophages.[26] The study was not controlled, and therefore unable to evaluate efficacy. However, we observed some initial signals related to enhanced fibrosis remodelling and liver function that warranted assessment in a randomised controlled trial as presented here.

Objectives

The primary objective of this phase 2 randomised controlled trial is to evaluate whether there is an improvement in liver function at 3 months in patients receiving autologous macrophage therapy compared to standard medical care.

The secondary objectives are to assess any improvement in markers of liver fibrosis, increased disease related quality of life, reduced liver related clinical events and prolonged transplant-free survival.

For peer review only

Trial Design

The MATCH trial is designed as a multicentre, open-label, parallel-group, phase 2, randomised controlled trial to compare autologous macrophage therapy with standard medical care in patients with compensated cirrhosis. Randomisation will be performed with a 1:1 allocation ratio and the primary outcome is the baseline to 90-day change in MELD score. Figure 1 provides an overview of trial pathway following randomisation to the respective arms. Initially, the proposed trial was designed to administer 3 infusions to those randomised to the treatment arm. It became apparent that it would not be acceptable or feasible to continue with 3 infusions due to the onerous commitment required of participants and the challenge to complete the trial within the proposed timeframe. Therefore, as a pragmatic approach, and in line with the phase 1 study, it was decided that a single infusion protocol should be adopted to simplify the participant journey and ensure adequate recruitment. This was agreed with the trial steering committee (TSC), sponsor and data monitoring committee (DMC).

Methods

Study oversight

The MATCH 0.1 trial is an investigator-led study, funded by the Medical Research Council (reference MR/M007588/1) and sponsored by ACCORD (Academic and Clinical Central Office for Research and Development for NHS Lothian/University of Edinburgh). Trial oversight is also provided by a trial steering committee (TSC) and data monitoring committee (DMC), who are impartial around aspects of study design and logistics but provide independent advice and interval safety analyses. The study started initially in 2016 and is likely to continue until late 2022. All study-related documents were designed by the trial team with input from ACCORD, an independent statistician and the Scottish National Blood Transfusion Service (SNBTS) team. The trial will be conducted according to the ethical principles of the Declaration of Helsinki 2013 and has been approved by Scotland A Research Ethics Committee (reference 15/SS/0121), NHS Lothian Research and Development department and the Medicine and

For peer review only

Health Care Regulatory Agency (MHRA-UK). The trial was registered in the International Standard Randomized Controlled Trial registry (ISRCTN10368050) and the European Clinical Trial Database (reference 2015-000963-15). Good Clinical Practice regulations will be followed and written informed consent will be obtained from all participants.

Study Setting

The MATCH trial is recruiting in 3 hepatology centres in Scotland: Royal Infirmary of Edinburgh (Tertiary Transplant Centre/Level 3 hepatology services), Ninewells Hospital, Dundee and Glasgow Royal Infirmary (both Level 2 hepatology centres). There are plans to potentially extend recruitment to include additional sites.

Patient and Public Involvement

There was no direct patient or public involvement groups involved in the study design. The overall study design was developed from previous experience of the investigators involved in the design and coordination of similar studies.

Eligibility Criteria (inclusions/exclusions)

Inclusion criteria

1. Aged between 18 and 75 years (inclusive) at time of screening
2. Aetiology: One or more of:
    a. *Alcohol Related Liver Disease* (No active alcohol misuse ≥6 calendar months prior to screening. Features of chronic liver disease with a compatible history of alcohol excess

For peer review only

(>80g/day), in the absence of other causes of chronic liver disease.

b. *Primary Biliary Cholangitis*

2 out of: Cholestatic LFTs

Positive anti-mitochondrial antibody (titre >1:40)

Compatible Liver Histology

(If already receiving Ursodeoxycholic Acid must be established on current dose >3 months prior to enrolment)

c. *Non-Alcoholic Fatty Liver Disease (NAFLD)*

Either: Histological evidence of hepatic steatosis in the absence of other liver diseases

Or:

Imaging compatible with NAFLD (e,g., fatty infiltration of liver) and one or more risk factors (e.g., elevated BMI, type-2 diabetes mellitus, hypertriglyceridemia, hypertension)

And:

The absence of significant alcohol consumption (<20g/day) and no evidence of other causes of chronic liver disease

d. *Cryptogenic Cirrhosis*

Diagnosis of cirrhosis un-attributable to any other cause

e. *Haemochromatosis*

Diagnosis made on basis of compatible biochemistry (transferrin saturation >60%,

For peer review only

ferritin >400), Genotype (homozygous C282Y or H63D compound heterozygote) or histology

f. *Alpha-1 antitrypsin deficiency*

Diagnosis based on compatible genetic, phenotypic or histological testing.

g. *Previous chronic Hepatitis C* (sustained viral response i.e. undetectable HCV RNA 24 weeks after treatment)

3. Diagnosis of cirrhosis – invasive or non-invasive criteria

Cirrhosis defined as Any of:

Biopsy-confirmed diagnosis of cirrhosis

Transient Elastography (TE) - ≥15kPa

Clinical and radiological features which in the opinion of the investigator correlate with a diagnosis of cirrhosis.

4. A MELD Score (Pre-2016) of ≥10 and ≤17 at screening visit

Exclusion criteria

Refusal or inability to give written informed consent to participate in the study.

i) Other causes of chronic liver disease/cirrhosis not included in the listed aetiologies

ii) Portal hypertensive haemorrhage; active episode of bleeding requiring hospitalisation in the last 3 months where varices have not been eradicated by endoscopic band ligation or TIPSS.

For peer review only

iii) Ascites unless, in the opinion of the investigator, is minimal and well controlled with no increase to diuretic therapy in the last 3 months.

iv) Hepatic encephalopathy; current or requiring hospitalisation for treatment in the last 3 months

v) HCC – uncertain cases to be discussed at the local hepatobiliary multidisciplinary team meeting (MDT). Dysplastic or indeterminate nodules to be excluded; regenerative or other nodules to be included at discretion of investigator.

vi) Previous diagnosis of HCC

vii) Previous organ transplant recipient

viii) Listed for liver transplantation

ix) Any situation that in the Investigators opinion may interfere with optimal study participation such as alcohol or drug abuse, domicile too distant from study site, potential non-compliance or inability to co-operate.

x) Presence of clinically relevant acute illness which may preclude on basis of safety.

xi) Presence or history of cancer with exception of adequately treated localised skin carcinoma, in-situ cervical cancer or solid malignancy excised in total, with no recurrence (5-year interval).

xii) Pregnancy or breastfeeding

Interventions

Participants who are randomised to the treatment arm will receive an infusion of the maximum achieved dose up to 1 x 10^9 (day 0). The apheresis product will be collected under the terms of the Human Tissue (Quality and Safety for Human Application) Regulations 2007 No. 1523 enacting the requirements of the EU Tissues and cells Directive (2004/23) and associated

For peer review only

Commission Directives at the Apheresis Unit (Royal Infirmary of Edinburgh, Edinburgh, UK). CD14+ monocytes will be isolated, and the macrophage cell product will be manufactured as previously described[27], in compliance with GMP regulations under the terms of the SNBTS MIA (IMP) licence at the SNBTS Cell Therapy Facility (Scottish Centre for Regenerative Medicine, Edinburgh, UK).

Each patient will be monitored closely during the infusion to identify potential hypersensitivity reactions and 4-hours post-infusion bloods to monitor for any evidence of macrophage activation syndrome (MAS). A total of 28 participants will be randomised to standard medical care and 28 to receive the cell infusion, allowing for original estimate of 5 dropouts from each arm. Additional safety data will be collected for the first infusion only for the first three patients randomised to the treatment arm. If it has not been possible to achieve $1x10^9$ macrophages, then the participants will be infused with the quantity obtained, with minimum concentration being 1.25×10^8 cells. This minimum cell concentration was derived from previous validation work and is stipulated as part of the product release criteria as designated by the MHRA.

Outcomes

Primary outcome measure

Model of End-Stage Liver Disease (MELD)

The Model for End-stage Liver Disease (MELD) was originally devised to predict survival in patients with complications of portal hypertension undergoing elective placement of trans-jugular intrahepatic portosystemic shunts (TIPSS). The algorithm is based on: creatinine, bilirubin and prothrombin ratio (PTr) and has been demonstrated to be superior to the Child-Turcotte-Pugh (CTP) score in predicting 3-month mortality among patients with end-stage liver disease (ESLD).[28] However, the MELD score has also been applied to predict survival in patients with cirrhosis with infections, variceal haemorrhage, and those with fulminant hepatic failure and alcoholic hepatitis.[29]

For peer review only

Secondary outcome measures

Transplant Free Interval

The number of participants in each of the 2 treatment arms who are transplant free at 12 months will be expressed as proportions and a binomial test will be used for the comparison of proportions between the treatment arm and the control arm. The difference in proportions will be presented along with the 95% confidence interval for the difference in the proportions.

The time to death or transplant will be presented using a Kaplan-Meier survival curve stratified by treatment and accompanied by a log-rank statistic comparing the two arms. Survival estimates with be presented by treatment arm at 3, 6, 9 and 12 months.

Non-Invasive Markers of Fibrosis

Changes in our secondary outcome measures over 90 days up to maximal 360 days as per schedule (Table 1), these include: serum Enhanced Liver Fibrosis (ELF) test (iQur, London, UK, serum Protein Fingerprint™ markers (Nordic Bioscience, Herlev, Denmark), hepatic Transient Elastography (TE; Echosens, Paris, France) and the United Kingdom Model for End-Stage Liver Disease (UKELD) score.

Enhanced Liver Fibrosis (ELF)

A standardised clinically validated immunoassay test measuring three serum biomarkers which have been shown to correlate to the level of liver fibrosis assessed by liver biopsy, comprising:

- Hyaluronic Acid (HA)
- Tissue Inhibitor of Metalloproteinase 1 (TIMP-1)
- Amino-terminal propeptide of type III procollagen (PIIINP)

For peer review only

The concentrations of each individual protein marker are combined in an algorithm which produces a composite score related to the level of liver fibrosis. The ELF score is a sensitive, specific, and validated method for the non-invasive assessment of hepatic fibrosis in mixed, HCV and NAFLD patient groups.[30]

Protein Fingerprint™ biomarkers

During extracellular matrix (ECM) turnover, proteolytically cleaved matrix degradation fragments, or neoepitopes, are released into the systemic circulation. Cleavage of each ECM protein by specific Matrix Metalloproteinases (MMPs) generates a unique neoepitope. These neoepitopes are more accurate diagnostic and prognostic markers for individual fibroproliferative diseases than their protein of origin. These novel serum biomarkers have been shown to identify patients with progressive fibrosis and permit monitoring of the response to antifibrotic therapy,[31] and also correlate with portal hypertension in patients with cirrhosis.[32]

Transient Elastography (Fibroscan)

Transient elastography is a non-invasive method for assessing liver fibrosis. Mild amplitude and low frequency vibrations (50Hz) are transmitted to the liver tissue, inducing an elastic shear wave that propagates through the underlying liver tissue. The velocity of the wave is directly related to tissue stiffness, considered as a surrogate of the amount of fibrotic tissue. This is expressed as a numerical value in kilopascals (kPa). It is reliable, reproducible with high intra- and inter-observer agreement and has been validated in most causes of chronic liver disease[33]

Chronic Liver Disease Quality of Life questionnaire (CLDQ)

The CLDQ is a liver specific questionnaire for measuring health related quality of life in participants with chronic liver disease. It is self-administered, takes approximately 10 minutes

For peer review only

For peer review only - http://bmjopen.bmj.com/site/about/guidelines.xhtml

to complete and is designed to reflect the two weeks prior to testing. If necessary, participants can request help to complete this.[34]

It includes 29 items divided into 6 quality of life domains: Abdominal symptoms, Fatigue, Systemic symptoms, Activity, Emotional function and Worry. These items are ranked on a 1 to 7 scale, providing a possible range of scores from 29 (worst quality of life) to 203 (best quality of life). The construct validity of the CLDQ was supported by a strong correlation with participant's global rating scores. It has been shown to be valid and has good test-retest reliability.[35–37]

United Kingdom End Stage Liver Disease (UKELD) score

The UKELD score is readily performed incorporating routine biochemical and haematological indices including bilirubin, albumin, ALT and INR. The UKELD score was developed by the UK Liver Transplant Units to predict transplant waiting list mortality.[38]

The score uses the parameters of Bilirubin (Bil), INR, Creatinine (Creat) and Sodium (Na) in the following algorithm:

*UKELD= [(5.395*ln(INR))+(1.485*ln(Creat)+(3.130*ln(Bil))-(81.565*ln(Na))]+435*

Magnetic resonance imaging and Magnetic Resonance Spectroscopy

Magnetic resonance imaging (MRI) and spectroscopy (MRS) provide methods for the non-invasive assessment of liver microstructure and function. MRI allows for imaging biomarkers to be determined using Liver*Multi*Scan.[39] Tissue microstructure will be investigated using clinically validated metrics. Fibrosis will be assessed by cT_1, iron content with T_2* and the amount of fat in the liver using proton density fat fraction. Organic phosphorus in the liver can be quantified with Phosphorus-31 (^{31}P) MRS[40] a more explorative technique. Using ^{31}P MRS energy metabolism may be investigated via ATP levels and cell membrane integrity by

measuring precursors and degradation products. The paired imaging of this study allows for the current utility of MRI to assess disease progression and treatment response to be evaluated

MRI data collected is exploratory and will be according to subgroup analysis: the only planned subgroup analysis is to present the primary outcome for the RCT by disease aetiology (ALD, NAFLD, other). MRI is performed at index visit 2 (or within 7 days) and again at primary outcome timepoint of 90days (+/- 7).

For peer review only

Sample Size/power calculation

To detect a difference in the baseline to 90-day change in MELD score of 1 standard deviation using a two-sided, two-sample test with a 5% level of significance, a sample size of 23 per group to detect the same level of difference with 90% power is required. All analyses will be carried out on an intention to treat basis, retaining participants in their randomised treatment groups irrespective of the treatment received. Adverse event data will be presented by treatment received.

The number of participants who do not adhere to the protocol is expected to be low. All protocol violations and ineligible participants will be recorded.

Recruitment

Identification of Potential Patients

Potential participants will be identified by their usual direct healthcare team. The treating physician will either introduce the individual to the trial team or ask permission for the trial team to contact them; this could be done through a dedicated invitation letter or a telephone call. The participant information sheet (PIS) will be provided and there will be an opportunity to ask questions. If they agree, a further visit will be scheduled to discuss trial enrolment. This will take place no less than 24 hours later.

For peer review only

Randomisation

Following confirmation of the participant meeting the eligibility criteria, a delegated member of the research team will enter minimal information (participant id, and aetiology) into an online randomisation system, produced for the study by Edinburgh Clinical Trials Unit (ECTU) to determine the treatment allocation. At randomisation, patients will be allocated a unique patient trial number and scheduled for treatment and follow up visits as detailed in the trial schedule.

Allocation

Participants will be assigned to receive either standard medical care or to receive a fresh dose of autologous MDMs at the maximum achievable dose, in a 1:1 ratio based on a minimisation algorithm using the key variable aetiology of disease (ALD, NAFLD, other.) To ensure the allocation is random, participants will be assigned to the group which minimises the imbalance with probability 0.8. If a participant falls into 2 or more strata, then the dominant aetiology (as determined by treating physician) will be used.

Blinding

Due to the nature of the intervention neither participants nor staff can be blinded to allocation of treatment. For some of the additional secondary outcomes we will maintain blinding of external assessors including those processing samples for ELF and protein fingerprint markers. Similarly, there is blinding of MRI physicists and external validation companies responsible for experimental MRI interpretation.

Data Collection

The Case Report Form (CRF) will be completed at set time points as per trial schedule. The CRF will be completed by the Investigator or an authorised member of the research team (as

For peer review only

delegated on the Site Signature and Delegation Log). The exception is the SAE Form which must be signed by the Investigator.

Data reported in each form should be consistent with the source data or the discrepancies should be explained. If information is not known, this must be clearly indicated in the form.

Completed CRFs submitted to the Clinical Research Facility will be reviewed by the Trial Co-ordinator. The data will be entered into an electronic database by designated members of the trial team.

Data Management

The following personal data will be collected as part of the research: Name, date of birth and CHI numbers (Community Health Index; a unique is a 10-character numeric identifier, allocated to each patient on first registration with the NHS system in Scotland). Personal data will be stored in locked cabinets by the research team at the Clinical Research Facilities at each site. Personal data will be stored for 30 years in keeping with the Blood Safety and Quality Regulations. The University of Edinburgh and NHS Lothian are joint data controllers along with any other entities involved in delivering the study that may be a data controller in accordance with applicable laws.

All Investigators and study site staff involved with this study must comply with the requirements of the appropriate data protection legislation (including where applicable the General Data Protection Regulation regarding the collection, storage, processing and disclosure of personal information. Access to personal information will be restricted to individuals from the research team treating the participants, representatives of the sponsor(s) and representatives of regulatory authorities.

For peer review only

Study data will be collected and managed using REDCap electronic data capture tools hosted at The University of Edinburgh. REDCap [41] (Research Electronic Data Capture) is a secure, web-based application designed to support data capture for research studies, providing: an intuitive interface for validated data entry; audit trails for tracking data manipulation and export procedures; automated export procedures for seamless data downloads to common statistical packages; and procedures for importing data from external sources.

Published results will not contain any personal data that could allow identification of individual participants.

Statistical Analysis Plan

The baseline to 90-day change in MELD score will be compared in the two treatment arms using a two-sample t-test or non-parametric equivalent as appropriate. MELD scores calculated for each participant throughout the trial will be used to calculate an area under the curve (AUC) and this will be compared across the groups using a two-sample t-test or non-parametric equivalent as appropriate. In the event of varying durations in the trial follow up, the average AUC per month will be used so that all participants have a comparable measurement.

Changes in secondary outcome measures (ELF score liver stiffness, CLDQ score, transplant-free survival, number of clinical events, UKELD score, blood parameters (bilirubin, albumin, ALT, INR)) over the 1-year study period will be presented graphically by dose. Similarly, these results will used to calculate an AUC for each participant and will be compared across the groups using a two-sample t-test or non-parametric equivalent as appropriate.

The only planned subgroup analysis is to present the primary outcome by disease aetiology (ALD, NAFLD, other). Primary data analysis will be conducted on participants who receive a single infusion versus control; the primary analysis will then be repeated to include those

For peer review only

subjects who receive more than one infusion (3 individuals). There are no plans for an interim analysis.

Data Monitoring

The trial will be coordinated by a Project Management Group, consisting of the grant holders (Chief Investigator and Principal Investigator in Edinburgh), a Trial Manager and coordinating nurse.

The Trial Manager will oversee the study and will be accountable to the Chief Investigator. The Trial Manager, or an authorised member of the research team, will be responsible for checking the CRFs for completeness, plausibility and consistency. Any queries will be resolved by the Investigator or delegated member of the trial team. A Delegation log will be prepared detailing the responsibilities of each member of staff working on the trial.

Safety assessments

The Investigator is responsible for the detection and documentation of events meeting the criteria and definitions detailed within the protocol (available on request). Full details of contraindications and side effects that have been reported following administration of the IMP can be found in the relevant Investigator's Brochure (IB).

Participants will be instructed to contact their Investigator at any time after consenting to join the trial if any symptoms develop. All adverse events (AE) that occur after joining the trial must be reported in detail in the Case Report Form (CRF) or AE form. In the case of an AE, the Investigator should initiate the appropriate treatment according to their medical judgement. Any AE events still present on day 360 will be confirmed and recorded as "ongoing" in the Case Report Form. If appropriate, these should be handed over to the participants' General Practitioner or direct care team.

For peer review only

The ACCORD Research Governance & QA Office is responsible for pharmacovigilance reporting on behalf of the co-sponsors (University of Edinburgh and NHS Lothian).

The ACCORD Research Governance & QA Office has a legal responsibility to notify the regulatory competent authority and relevant ethics committee (Research Ethics Committee (REC) that approved the trial). Fatal or life threatening Suspected Unexpected Serious Adverse Reactions (SUSARs) will be reported no later than 7 calendar days and all other SUSARs will be reported no later than 15 calendar days after ACCORD is first aware of the reaction.

ACCORD will inform Investigators at participating sites of all SUSARs and any other arising safety information.

An Annual Safety Report/Development Safety Update Report will be submitted, by ACCORD, to the regulatory authorities and RECs listing all SARs and SUSARs.

Monitoring and Oversight

An ACCORD Clinical Trials Monitor, or an appointed monitor will visit the Investigator site prior to the start of the study and during the course of the study if required, in accordance with the monitoring plan if required. Risk assessment will determine if audit, by the ACCORD QA group, is required. Details will be captured in an audit plan.

Discussion

MATCH is a randomised controlled trial designed to identify whether there is a measurable improvement in MELD score and also in relevant secondary clinical outcomes, HRQoL and non-invasive biomarkers following autologous macrophage therapy. It builds upon the safety and feasibility assessment of the earlier phase I trial. Recent FDA guidance on development of treatments for cirrhosis has indicated there are no acceptable surrogate endpoints (e.g.,

For peer review only

histological improvement) so our focus in this study is on clinically meaningful assessments such as liver function, survival and HRQoL rather than liver biopsy.

Previous clinical trials using mesenchymal stem cells (MSCs) across a range of aetiologies of liver disease have yielded mixed results. In trials which reported efficacy, the apparent benefit was transient, with no long-term improvement.[42,43]

One important rationale for utilising macrophages relates to the lack of efficacy of haematopoetic stem cells,[44] inherent challenges of using transplanted hepatocytes, and potential risk of introducing transplanted hepatocytes mesenchymal stem cells (MSCs) into a hostile host niche. Previous trials have demonstrated concerns around cellular engraftment and expansive potential of such approaches.

Preclinical studies undertaken by our group have administered macrophages via the portal vein, tail vein or intrasplenic route, but in our phase 1 trial we successfully used peripheral intravenous infusion which is safer and more convenient. Whilst there is no cell-tracking technique used in this trial to assess cell engraftment/durability, animal models and human case reports suggest that macrophages infused via either peripheral or central veins will transiently pass through the lungs, before engrafting in the liver and spleen.[45] However, hepatic artery or portal venous administration are considerably more invasive, with concerns regarding risk of bleeding and vessel injury[46], and problems related to reversal of portal flow/porto-systemic shunting or splanchnic vessel thrombosis .[47]

Through this trial, we aim to add to the collective knowledge of this potential new therapeutic modality for liver disease in this patient population who currently have limited treatment options. If effective, autologous macrophage cell therapy will improve clinical outcomes and enhance HRQoL in people with cirrhosis.

Following initial trial results, we expect that a further extended study will be necessary to determine longer term safety and the durability of treatment responses. Moreover, it is not yet clear whether patients may require repeat treatments to maximise efficacy.

We hope that this initial phase II trial will provide robust evidence to support and inform future trial design.

Ethics and dissemination

The trial will be conducted according to the ethical principles of the Declaration of Helsinki 2013 and has been approved by Scotland A Research Ethics Committee (reference 15/SS/0121), NHS Lothian Research and Development department and the Medicine and Health Care Regulatory Agency (MHRA-UK). The trial was registered in the International Standard Randomized Controlled Trial registry (ISRCTN10368050) and the European Clinical Trial Database (reference 2015-000963-15). Good Clinical Practice regulations will be followed and written informed consent will be obtained from all participants. Results will be disseminated through peer-reviewed publications, presented at conferences and published on clinicaltrials.gov. Ownership of the data arising from this study resides with the study team and their respective employers. The study team will follow the International Committee of Journal Editors (ICJME) guidelines. Requests for data access should be sent to the corresponding author (ORCID: 0000-0001-8368-1478).

For peer review only

Contributors: FM and SJF was responsible for the conceptualisation and design of the trial. PNB is study clinician and drafted manuscript and provided critical review of protocol. JAF aided manuscript preparation and critical appraisal. CG was responsible for statistical design. AG, CP, NWAM, ARF, MLT and JDMC were responsible reviewing sections around product manufacture. MM and TM provided manuscript review and critique. SIKS and DMM developed section on MRI imaging. NL and JFD provided critical appraisal of manuscript. All authors critically revised and approved the manuscript.

Conflict of Interests: PNB has received honoraria from Takeda. JAF has received consultancy fees for Ferring Pharmaceuticals, Macrophage Pharma, Aquilla BioMedical, Caldan Therapeutics, Cypralis Ltd, Third Rock Ventures, Rallybio, Narrow River Management, Gilde Healthcare, Guidepoint, Techspert.io and acted as advisory board member for: Novartis, Galecto Biotech, Tectonic Therapeutic and received research grant funding from Novartis and Intercept Pharmaceuticals. JFD has received honoraria and research grants from Gilead, AbbVie and MSD. JDMC and SJF are founders and scientific advisers to Resolution Therapeutics Ltd.

For peer review only

Bibliography

1. Asrani, S. K., Devarbhavi, H., Eaton, J. & Kamath, P. S. Burden of liver diseases in the world. *Journal of Hepatology* vol. 70 151–171 (2019).

2. Pimpin, L. *et al.* Burden of liver disease in Europe: Epidemiology and analysis of risk factors to identify prevention policies. *Journal of Hepatology* vol. 69 718–735 (2018).

3. Sepanlou, S. G. *et al.* The global, regional, and national burden of cirrhosis by cause in 195 countries and territories, 1990–2017: a systematic analysis for the Global Burden of Disease Study 2017. *The Lancet Gastroenterology and Hepatology* **5**, 245–266 (2020).

4. Younossi, Z. & Henry, L. Overall health-related quality of life in patients with end-stage liver disease. *Clinical Liver Disease* vol. 6 9–14 (2015).

5. Van Der Plas, S. M. *et al.* Generic and disease-specific health related quality of life of liver patients with various aetiologies: A survey. *Quality of Life Research* **16**, 375–388 (2007).

6. D'Amico, G., Garcia-Tsao, G. & Pagliaro, L. Natural history and prognostic indicators of survival in cirrhosis: A systematic review of 118 studies. *Journal of Hepatology* vol. 44 217–231 (2006).

7. Ratib, S., Fleming, K. M., Crooks, C. J., Aithal, G. P. & West, J. 1 and 5 year survival estimates for people with cirrhosis of the liver in England, 1998-2009: A large population study. *Journal of Hepatology* **60**, 282–289 (2014).

8. Arroyo, V. *et al.* The systemic inflammation hypothesis: Towards a new paradigm of acute decompensation and multiorgan failure in cirrhosis. *Journal of Hepatology* vol. 74 670–685 (2021).

9. Fallowfield, J. A., Jimenez-Ramos, M. & Robertson, A. Emerging synthetic drugs for the treatment of liver cirrhosis. *Expert Opinion on Emerging Drugs* 1–16 (2021) doi:10.1080/14728214.2021.1918099.

10. Neuberger, J. Liver transplantation in the United Kingdom. *Liver Transplantation* **22**, 1129–1135 (2016).

11. Ransford, R., Gunson, B., Mayer, D., Neuberger, J. & Christensen, E. Effect on outcome of the lengthening waiting list for liver transplantation. *Gut* vol. 47 441–443 (2000).

12. Moini, M., Schilsky, M. L. & Tichy, E. M. Review on immunosuppression in liver transplantation. *World Journal of Hepatology* vol. 7 1355–1368 (2015).

13. Forbes, S. J., Gupta, S. & Dhawan, A. Cell therapy for liver disease: From liver transplantation to cell factory. *Journal of Hepatology* vol. 62 S157–S169 (2015).

14. Dwyer, B. J., Macmillan, M. T., Brennan, P. N. & Forbes, S. J. Cell therapy for advanced liver diseases: Repair or rebuild. *Journal of Hepatology* (2020) doi:10.1016/j.jhep.2020.09.014.

15. Spahr, L. *et al.* Autologous Bone Marrow Mononuclear Cell Transplantation in Patients with Decompensated Alcoholic Liver Disease: A Randomized Controlled Trial. *PLoS ONE* **8**, (2013).

For peer review only

16. Newsome, P. N. *et al.* Granulocyte colony-stimulating factor and autologous CD133-positive stem-cell therapy in liver cirrhosis (REALISTIC): an open-label, randomised, controlled phase 2 trial. *The Lancet Gastroenterology and Hepatology* **3**, 25–36 (2018).

17. Tacke, F. Functional role of intrahepatic monocyte subsets for the progression of liver inflammation and liver fibrosis in vivo. *Fibrogenesis & Tissue Repair* **5**, S27 (2012).

18. Ramachandran, P. *et al.* Differential Ly-6C expression identifies the recruited macrophage phenotype, which orchestrates the regression of murine liver fibrosis. *Proceedings of the National Academy of Sciences* **109**, E3186–E3195 (2012).

19. SJ, F. & N, R. Preparing the ground for tissue regeneration: from mechanism to therapy. *Nature medicine* **20**, 857–869 (2014).

20. Thomas, J. A. *et al.* Macrophage therapy for murine liver fibrosis recruits host effector cells improving fibrosis, regeneration, and function. *Hepatology* **53**, 2003–2015 (2011).

21. Thomas, J. A. *et al.* Macrophage therapy for murine liver fibrosis recruits host effector cells improving fibrosis, regeneration, and function. *Hepatology* **53**, 2003–2015 (2011).

22. Ramachandran, P. *et al.* Differential Ly-6C expression identifies the recruited macrophage phenotype, which orchestrates the regression of murine liver fibrosis. *Proceedings of the National Academy of Sciences of the United States of America* **109**, E3186–E3195 (2012).

23. Bird, T. G. *et al.* Bone marrow injection stimulates hepatic ductular reactions in the absence of injury via macrophage-mediated TWEAK signaling. *Proceedings of the National Academy of Sciences of the United States of America* **110**, 6542–6547 (2013).

24. Moore, J. K. *et al.* Phenotypic and functional characterization of macrophages with therapeutic potential generated from human cirrhotic monocytes in a cohort study. *Cytotherapy* **17**, 1604–1616 (2015).

25. P, S. L. *et al.* Alternatively activated macrophages promote resolution of necrosis following acute liver injury. *Journal of hepatology* **73**, 349–360 (2020).

26. Moroni, F. *et al.* Safety profile of autologous macrophage therapy for liver cirrhosis. *Nature Medicine* **25**, 1560–1565 (2019).

27. Fraser, A. R. *et al.* Development, functional characterization and validation of methodology for GMP-compliant manufacture of phagocytic macrophages: A novel cellular therapeutic for liver cirrhosis. *Cytotherapy* **19**, 1113–1124 (2017).

28. Cuomo, O., Perrella, A. & Arenga, G. Model for End-Stage Liver Disease (MELD) Score System to Evaluate Patients With Viral Hepatitis on the Waiting List: Better Than the Child-Turcotte-Pugh (CTP) System? *Transplantation Proceedings* **40**, 1906–1909 (2008).

29. Kamath, P. S. & Kim, W. R. The Model for End-stage Liver Disease (MELD). *Hepatology* vol. 45 797–805 (2007).

30. Rosenberg, W. M. C. *et al.* Serum markers detect the presence of liver fibrosis: A cohort study. *Gastroenterology* **127**, 1704–1713 (2004).

For peer review only

31. Leeming, D. J. *et al.* Pro-C5, a marker of true type V collagen formation and fibrillation, correlates with portal hypertension in patients with alcoholic cirrhosis. *Scandinavian Journal of Gastroenterology* **50**, 584–592 (2015).

32. Karsdal, M. A. *et al.* Review article: The efficacy of biomarkers in chronic fibroproliferative diseases - Early diagnosis and prognosis, with liver fibrosis as an exemplar. *Alimentary Pharmacology and Therapeutics* vol. 40 233–249 (2014).

33. Fraquelli, M. *et al.* Reproducibility of transient elastography in the evaluation of liver fibrosis in patients with chronic liver disease. *Gut* **56**, 968–973 (2007).

34. Younossi, Z. M., Guyatt, G., Kiwi, M., Boparai, N. & King, D. Development of a disease specific questionnaire to measure health related quality of life in patients with chronic liver disease. *Gut* **45**, 295–300 (1999).

35. Lam, E. T. P., Lam, C. L. K., Lai, C. L., Yuen, M. F. & Fong, D. Y. T. Psychometrics of the chronic liver disease questionnaire for Southern Chinese patients with chronic hepatitis B virus infection. *World Journal of Gastroenterology* **15**, 3288–3297 (2009).

36. Kallman, J. *et al.* Fatigue and health-related quality of life (HRQL) in chronic hepatitis C virus infection. *Digestive Diseases and Sciences* **52**, 2531–2539 (2007).

37. Chawla, K. S. *et al.* Reliability and validity of the Chronic Liver Disease Questionnaire (CLDQ) in adults with non-alcoholic steatohepatitis (NASH). *BMJ Open Gastroenterology* **3**, (2016).

38. Barber, K. *et al.* Elective liver transplant list mortality: Development of a United Kingdom end-stage liver disease score. *Transplantation* **92**, 469–476 (2011).

39. Pavlides, M. *et al.* Multiparametric magnetic resonance imaging predicts clinical outcomes in patients with chronic liver disease. *Journal of Hepatology* **64**, 308–315 (2016).

40. Valkovič, L., Chmelík, M. & Krššák, M. In-vivo 31P-MRS of skeletal muscle and liver: A way for non-invasive assessment of their metabolism. *Analytical Biochemistry* **529**, 193–215 (2017).

41. Harris, P. A. *et al.* Research electronic data capture (REDCap)-A metadata-driven methodology and workflow process for providing translational research informatics support. *Journal of Biomedical Informatics* **42**, 377–381 (2009).

42. Zekri, A.-R. N. *et al.* The impact of repeated autologous infusion of haematopoietic stem cells in patients with liver insufficiency. *Stem Cell Research & Therapy 2015 6:1* **6**, 1–14 (2015).

43. M, M. *et al.* Randomized placebo-controlled trial of mesenchymal stem cell transplantation in decompensated cirrhosis. *Liver international : official journal of the International Association for the Study of the Liver* **33**, 1490–1496 (2013).

44. King, A. *et al.* REpeated AutoLogous Infusions of STem cells In Cirrhosis (REALISTIC): a multicentre, phase II, open-label, randomised controlled trial of repeated autologous infusions of granulocyte colony-stimulating factor (GCSF) mobilised CD133+ bone marrow stem cells in patients with cirrhosis. A study protocol for a randomised controlled trial. doi:10.1136/bmjopen-2015-007700.

45. Bird, T. G. *et al.* Bone marrow injection stimulates hepatic ductular reactions in the absence of injury via macrophage-mediated TWEAK signaling. *Proceedings of the National Academy of Sciences* **110**, 6542–6547 (2013).

For peer review only

46. BG, C. *et al.* Bone marrow mononuclear cell therapy for patients with cirrhosis: a Phase 1 study. *Liver international : official journal of the International Association for the Study of the Liver* **31**, 391–400 (2011).

47. Dwyer, B. J., Macmillan, M. T., Brennan, P. N. & Forbes, S. J. Cell therapy for advanced liver diseases: Repair or rebuild. *Journal of Hepatology* vol. 74 185–199 (2021).

Fig 1: Schematic of Trial Timeline

For peer review only

			Treatment group				Control group					
	**Screening**	**Randomisation**	**Apheresis**	**Cell Infusion**	**Safety Visit**	**Safety Visit**	**Research sample**	**Follow-up**	**Follow-up**	**Follow-up**	**Follow-up**	**Follow-up**
	**Visit 1a**	**Visit 1b**	**Visit 2a Within 7±4 days of Visit 1b**	**Visit 2b 7>10days after apheresis (Day 0)**	**Visit 2c (Day 7)**	**Visit 2d (Day 14)**	**Visit 2 (day 7±4days from visit 1b)**	**Visit 3 (Day 28±4 days)**	**Visit 4 (Day 56±4 days)**	**Visit 5 (Day 90±7 days)**	**Visit 6 (Day 180±7 days)**	**Visit 7 (Day 360±7 days)**
Informed consent	X											
Clinical Assessment	X	X	X	X	X	X	X	X	X	X	X	X
Vital Signs	X	X	X	X	X	X		X	X	X	X	X
Screening Blood Tests	X											
ECG	X											
Standard Blood Tests	X	X		X	X	X		X	X	X	X	X
Research Bloods* **			X		X	X	X	X	X	X	X	X
Mandatory Microbiology	X		X									
Ferritin	X			X##	X	X						
Triglyceride	X			X##	X	X						
Pre-infusion blood tests				X								
MELD/UKELD	X	X		X	X	X		X	X	X	X	X
Pregnancy test	X*	X*		X*	X**							X*
Abdominal USS	X[1]										X[1]	X[1]
Fibroscan	X									X	X	X
ELF Panel	X							X	X	X	X	X
Protein Fingerprint™	X[1]							X[1]		X[1]		
CLDQ		X								X	X	X
31P MRS MRI#	X***									X		
Adverse Events	X	X	X	X	X	X	X	X	X	X	X	X
Clinical Events	X	X	X	X	X	X	X	X	X	X	X	X
Concomitant Medication	X	X	X	X	X	X	X	X	X	X	X	X

*women of child bearing age only ** If test not carried out at previous visit *** If pass screen & before visit 2b [1]fasted visit #RIE patients only ##obtain before discharge*

Table 1: Trial Assessment Schedule

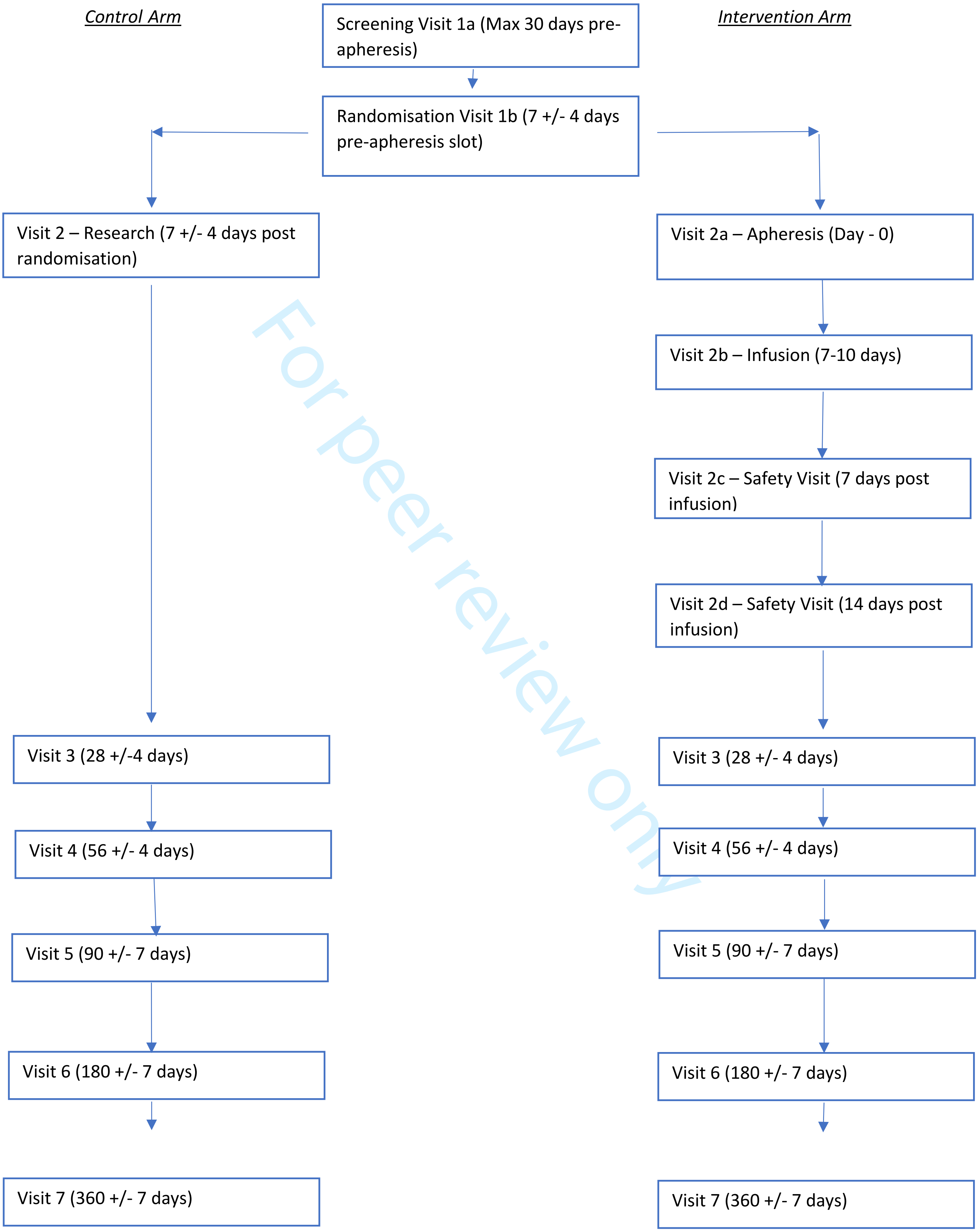

For peer review only

Reporting checklist for protocol of a clinical trial.

Based on the SPIRIT guidelines.

Instructions to authors

Complete this checklist by entering the page numbers from your manuscript where readers will find each of the items listed below.

Your article may not currently address all the items on the checklist. Please modify your text to include the missing information. If you are certain that an item does not apply, please write "n/a" and provide a short explanation.

Upload your completed checklist as an extra file when you submit to a journal.

In your methods section, say that you used the SPIRITreporting guidelines, and cite them as:

Chan A-W, Tetzlaff JM, Gøtzsche PC, Altman DG, Mann H, Berlin J, Dickersin K, Hróbjartsson A, Schulz KF, Parulekar WR, Krleža-Jerić K, Laupacis A, Moher D. SPIRIT 2013 Explanation and Elaboration: Guidance for protocols of clinical trials. BMJ. 2013;346:e7586

		Reporting Item	Page Number
**Administrative information**			
Title	#1	Descriptive title identifying the study design, population, interventions, and, if applicable, trial acronym	1
Trial registration	#2a	Trial identifier and registry name. If not yet registered,	2

For peer review only

		name of intended registry	
Trial registration: data set	#2b	All items from the World Health Organization Trial Registration Data Set	N/A
Protocol version	#3	Date and version identifier	3
Funding	#4	Sources and types of financial, material, and other support	3
Roles and responsibilities: contributorship	#5a	Names, affiliations, and roles of protocol contributors	26
Roles and responsibilities: sponsor contact information	#5b	Name and contact information for the trial sponsor	9
Roles and responsibilities: sponsor and funder	#5c	Role of study sponsor and funders, if any, in study design; collection, management, analysis, and interpretation of data; writing of the report; and the decision to submit the report for publication, including whether they will have ultimate authority over any of these activities	9
Roles and responsibilities: committees	#5d	Composition, roles, and responsibilities of the coordinating centre, steering committee, endpoint adjudication committee, data management team, and other individuals or groups overseeing the trial, if applicable (see Item 21a for data monitoring committee)	9

Introduction

For peer review only

Background and rationale	#6a	Description of research question and justification for undertaking the trial, including summary of relevant studies (published and unpublished) examining benefits and harms for each intervention	5-8
Background and rationale: choice of comparators	#6b	Explanation for choice of comparators	N/A
Objectives	#7	Specific objectives or hypotheses	9
Trial design	#8	Description of trial design including type of trial (eg, parallel group, crossover, factorial, single group), allocation ratio, and framework (eg, superiority, equivalence, non-inferiority, exploratory)	7,19
**Methods: Participants, interventions, and outcomes**			
Study setting	#9	Description of study settings (eg, community clinic, academic hospital) and list of countries where data will be collected. Reference to where list of study sites can be obtained	9, 19
Eligibility criteria	#10	Inclusion and exclusion criteria for participants. If applicable, eligibility criteria for study centres and individuals who will perform the interventions (eg,	10, 11, 12

For peer review only

		surgeons, psychotherapists)	
Interventions: description	#11a	Interventions for each group with sufficient detail to allow replication, including how and when they will be administered	12
Interventions: modifications	#11b	Criteria for discontinuing or modifying allocated interventions for a given trial participant (eg, drug dose change in response to harms, participant request, or improving / worsening disease)	N/A
Interventions: adherance	#11c	Strategies to improve adherence to intervention protocols, and any procedures for monitoring adherence (eg, drug tablet return; laboratory tests)	30
Interventions: concomitant care	#11d	Relevant concomitant care and interventions that are permitted or prohibited during the trial	N/A
Outcomes	#12	Primary, secondary, and other outcomes, including the specific measurement variable (eg, systolic blood pressure), analysis metric (eg, change from baseline, final value, time to event), method of aggregation (eg, median, proportion), and time point for each outcome. Explanation of the clinical relevance of chosen efficacy and harm outcomes is strongly recommended	14-17
Participant timeline	#13	Time schedule of enrolment, interventions (including any run-ins and washouts), assessments, and visits for participants. A schematic diagram is highly recommended (see Figure)	18

For peer review only

Sample size	#14	Estimated number of participants needed to achieve study objectives and how it was determined, including clinical and statistical assumptions supporting any sample size calculations	19
Recruitment	#15	Strategies for achieving adequate participant enrolment to reach target sample size	1920
**Methods: Assignment of interventions (for controlled trials)**			
Allocation: sequence generation	#16a	Method of generating the allocation sequence (eg, computer-generated random numbers), and list of any factors for stratification. To reduce predictability of a random sequence, details of any planned restriction (eg, blocking) should be provided in a separate document that is unavailable to those who enrol participants or assign interventions	20
Allocation concealment mechanism	#16b	Mechanism of implementing the allocation sequence (eg, central telephone; sequentially numbered, opaque, sealed envelopes), describing any steps to conceal the sequence until interventions are assigned	20
Allocation: implementation	#16c	Who will generate the allocation sequence, who will enrol participants, and who will assign participants to interventions	20

For peer review only

Blinding (masking)	#17a	Who will be blinded after assignment to interventions (eg, trial participants, care providers, outcome assessors, data analysts), and how	20
Blinding (masking): emergency unblinding	#17b	If blinded, circumstances under which unblinding is permissible, and procedure for revealing a participant's allocated intervention during the trial	N/A
**Methods: Data collection, management, and analysis**			
Data collection plan	#18a	Plans for assessment and collection of outcome, baseline, and other trial data, including any related processes to promote data quality (eg, duplicate measurements, training of assessors) and a description of study instruments (eg, questionnaires, laboratory tests) along with their reliability and validity, if known. Reference to where data collection forms can be found, if not in the protocol	20, 21
Data collection plan: retention	#18b	Plans to promote participant retention and complete follow-up, including list of any outcome data to be collected for participants who discontinue or deviate from intervention protocols	20, 21
Data management	#19	Plans for data entry, coding, security, and storage, including any related processes to promote data quality (eg, double data entry; range checks for data values).	21

For peer review only

		Reference to where details of data management procedures can be found, if not in the protocol	
Statistics: outcomes	#20a	Statistical methods for analysing primary and secondary outcomes. Reference to where other details of the statistical analysis plan can be found, if not in the protocol	22
Statistics: additional analyses	#20b	Methods for any additional analyses (eg, subgroup and adjusted analyses)	22
Statistics: analysis population and missing data	#20c	Definition of analysis population relating to protocol non-adherence (eg, as randomised analysis), and any statistical methods to handle missing data (eg, multiple imputation)	N/A
**Methods: Monitoring**			
Data monitoring: formal committee	#21a	Composition of data monitoring committee (DMC); summary of its role and reporting structure; statement of whether it is independent from the sponsor and competing interests; and reference to where further details about its charter can be found, if not in the protocol. Alternatively, an explanation of why a DMC is not needed	9
Data monitoring: interim analysis	#21b	Description of any interim analyses and stopping guidelines, including who will have access to these interim results and make the final decision to terminate the trial	9, 23
Harms	#22	Plans for collecting, assessing, reporting, and managing solicited and spontaneously reported adverse events and other unintended effects of trial interventions or trial	20, 21, 23

For peer review only

		conduct	
Auditing	#23	Frequency and procedures for auditing trial conduct, if any, and whether the process will be independent from investigators and the sponsor	23
**Ethics and dissemination**			
Research ethics approval	#24	Plans for seeking research ethics committee / institutional review board (REC / IRB) approval	2, 9
Protocol amendments	#25	Plans for communicating important protocol modifications (eg, changes to eligibility criteria, outcomes, analyses) to relevant parties (eg, investigators, REC / IRBs, trial participants, trial registries, journals, regulators)	N/A
Consent or assent	#26a	Who will obtain informed consent or assent from potential trial participants or authorised surrogates, and how (see Item 32)	9, 12, 23
Consent or assent: ancillary studies	#26b	Additional consent provisions for collection and use of participant data and biological specimens in ancillary studies, if applicable	N/A
Confidentiality	#27	How personal information about potential and enrolled participants will be collected, shared, and maintained in order to protect confidentiality before, during, and after the trial	20, 21
Declaration of	#28	Financial and other competing interests for principal	25

For peer review only

interests		investigators for the overall trial and each study site	
Data access	#29	Statement of who will have access to the final trial dataset, and disclosure of contractual agreements that limit such access for investigators	20, 21, 24
Ancillary and post trial care	#30	Provisions, if any, for ancillary and post-trial care, and for compensation to those who suffer harm from trial participation	N/A
Dissemination policy: trial results	#31a	Plans for investigators and sponsor to communicate trial results to participants, healthcare professionals, the public, and other relevant groups (eg, via publication, reporting in results databases, or other data sharing arrangements), including any publication restrictions	2
Dissemination policy: authorship	#31b	Authorship eligibility guidelines and any intended use of professional writers	N/A
Dissemination policy: reproducible research	#31c	Plans, if any, for granting public access to the full protocol, participant-level dataset, and statistical code	N/A

Appendices

Informed consent materials	#32	Model consent form and other related documentation given to participants and authorised surrogates	N/A
Biological specimens	#33	Plans for collection, laboratory evaluation, and storage of biological specimens for genetic or molecular analysis in the current trial and for future use in ancillary studies, if applicable	N/A

For peer review only

The SPIRIT Explanation and Elaboration paper is distributed under the terms of the Creative Commons Attribution License CC-BY-NC. This checklist was completed on 05. May 2021 using https://www.goodreports.org/, a tool made by the EQUATOR Network in collaboration with Penelope.ai

For peer review only
